# Exploring the Anticancer Potential of Semisynthetic Derivatives of 7α-Acetoxy-6β-hydroxyroyleanone from *Plectranthus* sp.: An In Silico Approach

**DOI:** 10.3390/ijms25084529

**Published:** 2024-04-20

**Authors:** Anna Merecz-Sadowska, Vera M. S. Isca, Przemysław Sitarek, Tomasz Kowalczyk, Magdalena Małecka, Karolina Zajdel, Hanna Zielińska-Bliźniewska, Mariusz Jęcek, Patricia Rijo, Radosław Zajdel

**Affiliations:** 1 Department of Economic and Medical Informatics, University of Lodz, 90-214 Lodz, Poland; mariusz.jecek@uni.lodz.pl (M.J.); radoslaw.zajdel@uni.lodz.pl (R.Z.); 2 Department of Allergology and Respiratory Rehabilitation, Medical University of Lodz, 90-725 Lodz, Poland; hanna.zielinska-blizniewska@umed.lodz.pl; 3 Center for Research in Biosciences & Health Technologies (CBIOS), Universidade Lusófona de Humanidades e Tecnologias, 1749-024 Lisboa, Portugal; vara.isca@ulusofona.pt; 4 Department of Medical Biology, Medical University of Lodz, Muszynskiego 1, 90-151 Lodz, Poland; przemyslaw.sitarek@umed.lodz.pl; 5 Department of Molecular Biotechnology and Genetics, Faculty of Biology and Environmental Protection, University of Lodz, Banacha 12/16, 90-237 Lodz, Poland; tomasz.kowalczyk@biol.uni.lodz.pl; 6 Department of Physical Chemistry, Faculty of Chemistry, University of Lodz, Pomorska 163/165, 90-236 Lodz, Poland; magdalena.malecka@chemia.uni.lodz.pl; 7 Department of Medical Informatics and Statistics, Medical University of Lodz, 90-645 Lodz, Poland; karolina.smigiel@umed.lodz.pl; 8 Instituto de Investigação do Medicamento (iMed.ULisboa), Faculdade de Farmácia, Universidade de Lisboa, 1649-003 Lisboa, Portugal

**Keywords:** 7α-acetoxy-6β-hydroxyroyleanone, *Plectranthus grandidentatus*, anticancer activity

## Abstract

The diterpene 7α-acetoxy-6β-hydroxyroyleanone isolated from *Plectranthus grandidentatus* demonstrates promising antibacterial, anti-inflammatory and anticancer properties. However, its bioactivity may be enhanced via strategic structural modifications of such natural products through semisynthesis. The anticancer potential of 7α-acetoxy-6β-hydroxyroyleanone and five derivatives was analyzed in silico via the prediction of chemicals absorption, distribution, metabolism, excretion, and toxicity (ADMET), quantum mechanical calculations, molecular docking and molecular dynamic simulation. The protein targets included regulators of apoptosis and cell proliferation. Additionally, network pharmacology was used to identify potential targets and signaling pathways. Derivatives 7α-acetoxy-6β-hydroxy-12-*O*-(2-fluoryl)royleanone and 7α-acetoxy-6β-(4-fluoro)benzoxy-12-*O*-(4-fluoro)benzoylroyleanone achieved high predicted binding affinities towards their respective protein panels, with stable molecular dynamics trajectories. Both compounds demonstrated favorable ADMET parameters and toxicity profiles. Their stability and reactivity were confirmed via geometry optimization. Network analysis revealed their involvement in cancer-related pathways. Our findings justify the inclusion of 7α-acetoxy-6β-hydroxy-12-*O*-(2-fluoryl)royleanone and 7α-acetoxy-6β-(4-fluoro)benzoxy-12-*O*-(4-fluoro)benzoylroyleanone in in vitro analyses as prospective anticancer agents. Our binding mode analysis and stability simulations indicate their potential as selective inhibitors. The data will guide studies into their structure optimization, enhancing efficacy and drug-likeness.

## 1. Introduction

Cancer remains one of the leading causes of death worldwide, second only to cardiovascular diseases [1]. According to predictions, the number of annual cancer deaths will increase from 7.1 million in 2002 to 11.5 million in 2030 [2]. The most prevalent cancer types differ between sexes: the dominant types in men include cancers of the lung, liver, colorectal, prostate and stomach, while in women, cancer mainly affects the breasts, colorectal, cervical, thyroid and lungs. Recent analyses indicate that around 30–40% of the risk of cancer can be attributed to factors such as tobacco use, alcohol consumption and obesity [3]. Additionally, population-based genomic studies emphasize the role of non-mutagenic agents as promoters of carcinogenesis [4]. Conventional cancer treatment relies heavily on surgical resections, chemotherapy and radiotherapy; however, these are associated with numerous side effects and insufficient selectivity, and there is great interest in identifying novel, natural and synthetic compounds with potential anticancer properties [4,5].

Plants are a valuable source of biologically active metabolites, providing not only essential nutrients but also compounds beneficial for human health and the treatment of diseases [6,7]. They contain a wide variety of specialized metabolites that are used in pharmaceutical, cosmetic and agricultural industries [8]. The genus *Plectranthus* encompasses around 300 species with established ethnobotanical and medicinal uses. Approximately 62 *Plectranthus* species are used in medicinal applications, as are ornamental plants [9,10]. Their documented applications include the treatment of digestive, dermatological, infectious and respiratory conditions [11,12]. *Plectranthus* is also a rich source of abietane diterpenoids, characterized by a tricyclic abietane carbon skeleton substituted with various functional groups [13,14]. Abietanes demonstrate a wide range of antibacterial, anti-inflammatory, antioxidant, anticancer and antidiabetic properties [15,16,17,18].

A comprehensive review by Abdel-Mogib et al. [19] highlights the phytochemical diversity of the genus *Plectranthus* based on the data in the literature from up to 1999. The authors reported the isolation of approximately 140 diterpenoids, primarily highly modified abietanes such as royleanones, spirocoleons, quinone methides and acylhydroquinones. Interestingly, no iridoid glycosides or clerodane diterpenoids, common in other *Lamiaceae* genera, were found in *Plectranthus*. This genus is also rich in essential oils containing mono- and sesquiterpenoids. Additionally, minor amounts of triterpenoids, flavonoids, and long-chain alkylphenols with potential chemotaxonomic significance were identified. The review underscores the structural diversity of *Plectranthus* metabolites and their potential therapeutic applications.

Depending on the introduced structural alterations, the activity of natural compounds may be enhanced or attenuated via semisynthesis [20,21]. The anticancer and cytotoxic potentials of these derivatives can be evaluated using various in silico methodologies. These computational approaches allow for the high-throughput assessment of prospective compound libraries and the optimization of lead structures prior to chemical synthesis [22]. Most in silico techniques are used to predict pharmacokinetic properties, toxicity profiles and overall drug-likeness, while docking simulations can be used to more specifically predict and score the binding modes and affinities between small-molecule ligands and their biological targets. Together, these computational methods can guide subsequent in vitro studies by prioritizing promising derivatives and predicting their interactions with specific protein residues. In addition, computational chemistry has enabled the extensive use of in silico analyses focused on the discovery of novel drug candidates with improved safety profiles [23,24,25].

The aim of this study is to present a comprehensive in silico evaluation of the anticancer potential of five semisynthetic derivatives of the abietane diterpene 7α-acetoxy-6β-hydroxyroyleanone. The study comprised a number of analyses. Pharmacokinetic parameters, such as drug-likeness and toxicity profiles, were assessed with the SwissADME, STOPTox and Osiris platforms. Equilibrium geometries were optimized and electronic properties calculated using the density functional theory methodology. Molecular docking simulations were performed against a panel of protein targets involved in carcinogenesis. The stability of protein–ligand complexes over time was determined using molecular dynamics simulations. Additionally, network pharmacology approaches were utilized to construct protein–protein interaction networks and identify signaling pathways. This multi-tiered in silico approach allowed for the efficient screening and selection of the most promising candidates from a focused library for further in vitro analyses.

## 2. Results

### 2.1. Characterization of the Synthesized Compounds

**Figure 1:**
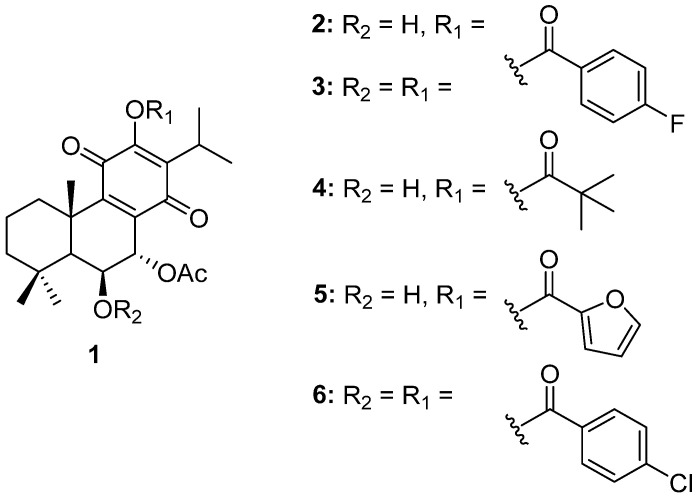
Molecule **1** and derivatives (**2**–**6**) synthetized from 1 (Roy).

The chemical structures of molecule **1** (Roy) and its semi-synthetic derivatives (**2**–**6**) are presented in Figure 1.

The structures of the synthesized 7α-acetoxy-6β-hydroxyroyleanone derivatives (**2**–**6**) were confirmed using Fourier-transform infrared spectroscop (FTIR), nuclear magnetic resonance (NMR) and high-resolution mass spectrometry (HRMS) analyses. 

The ^1^H NMR spectra of compounds **2**, **4**, and **5** displayed additional signals corresponding to those of the introduced ester groups at the 12-position, while preserving the signals of the parent compound (**1**). For compounds **3** and **6**, the ^1^H NMR spectra showed a downfield shift of the H-6 signal compared with that of compound 1, confirming the esterification of the 6-OH group. The ^13^C NMR spectra of all derivatives exhibited additional carbonyl carbon signals, further supporting the presence of the ester functionalities.

HRMS analysis provided molecular ion peaks, [M + H]+ or [M + Na]+, consistent with the calculated masses of the esterified products, confirming their molecular formulas and the success of the esterification reactions. 

The original spectra are provided in the Supplementary Information.

### 2.2. ADMET and Drug-Likeness Analysis Results

The physicochemical properties of the screened compounds are presented in Supplementary Table S1 (SwissAdme server). The molecular weights (MW) of the selected compounds ranged from 348.43 to 634.66 g/mol. Compounds **2**, **3** and **6** have a molecular weight higher than 500 g/mol. 

The lipophilicity properties of the screened compounds are presented in Supplementary Table S2 (SwissAdme server) as consensus LogP values. The lipophilicity of the tested compounds ranged from 2.31 to 6.40, with that of **3** and **6** being above 5; this indicates that these molecules may offer promise as candidates for oral medications. Lipophilicity is an important physicochemical property in pharmacokinetics and drug discovery.

The water solubility properties of the screened compounds are presented in Supplementary Table S3 (SwissAdme server). The screened compounds exhibit a range of water solubility categories, from moderately to poorly soluble, indicating that efforts should be taken to enhance solubility during the formulation process.

The pharmacokinetic parameters of the screened compounds are presented in Supplementary Table S4 (SwissAdme server). Briefly, **1**, **2**, **4** and **5** demonstrated high gastrointestinal absorption. The blood-brain barrier (BBB) permeability potential was not predicted for all compounds. All the compounds showed the potential to be substrates of P-gp. Cytochrome P450 (CYP) inhibition was noted for compounds **2** and **5** (for two isoforms), and for compounds **1**, **3**, **4** and **6** (for one isoform). 

The drug-likeness, medicinal chemistry and lead-likeness parameters are presented in Supplementary Table S5 (SwissAdme server). Almost all tested compounds violated at least one of the five filters (Lipinski, Ghose, Veber, Egan, and Muegge); however, **1** did not violate any. The computed bioavailability score for all the compounds placed them within the 56% probability class. All the compounds showed at least one pan-assay interference compounds (PAINS) or BRENK alerts and lead-likeness violation.

### 2.3. Toxicity Prediction and Molecular Property Results

Rat oral acute toxicity (LD50) expressed in mg/kg, along with the predicted toxicity classes (I–VI) and the corresponding prediction accuracy percentages, are presented in Table 1 (ProTox-II server). Compounds **2**–**6** exhibit the highest toxicity, i.e., class III (50 < LD50 ≤ 300), indicating potential toxicity risks upon oral exposure. Compound **1** demonstrated the lowest toxicity, i.e., class IV (300 < LD50 ≤ 2000), representing compounds most likely to cause harm following oral exposure. None of the compounds were classified as belonging to class 1 (LD50 ≤ 5) and 2 (5 < LD50 ≤ 50), i.e., potentially lethal. Furthermore, no compounds fell within class 5 (2000 < LD50 ≤ 5000) or class 6 (LD50 > 5000); the former identifies compounds that are less likely to be harmful when orally exposed, while the latter designates compounds as non-toxic.

The toxicity data for the screened compounds are presented in Supplementary Table S6 (ProTox-II server). None of the compounds were found to be hepatotoxic. All except **4** were found to be carcinogenic, with probability scores ranging from 0.50 to 0.58. Additionally, all compounds demonstrated immunotoxic activity with probability scores ranging from 0.84 to 0.99. However, no compounds were found to be mutagenic or cytotoxic. The predicted Tox21-nuclear receptor signaling pathway parameters aryl hydrocarbon receptor (AhR), androgen receptor (AR), androgen receptor ligand binding domain (AR-LBD), aromatase, estrogen receptor alpha (ER), estrogen receptor ligand binding domain (ER-LBD) and peroxisome proliferator activated receptor gamma (PPAR-Gamma) are presented in Supplementary Table S7 (ProTox-II server). 

The predicted Tox21 stress response pathway parameters nuclear factor (erythroid-derived 2)-like 2/antioxidant responsive element (nrf2/ARE), heat shock factor response element (HSE), mitochondrial membrane potential (MMP), phosphoprotein (Tumor Supressor) p53 and ATPase family AAA domain-containing protein 5 (ATAD5) are given in Supplementary Table S8 (ProTox-II server). Briefly, **3**, **5** and **6** exhibited activity in interacting with the mitochondrial membrane potential, with probability scores of 0.52, 0.50 and 0.56, respectively.

The predicted acute toxicity, including inhalation, oral and dermal toxicity, as well as eye irritation and corrosion, skin sensitization and skin irritation and corrosion are presented in Supplementary Table S9 (StopTox server). The compounds did not exhibit any form of toxicity upon exposure.

The predicted mutagenic, tumorigenic and irritant potential, and reproductive effectivity are presented in Supplementary Table S10 (OSIRIS server). All compounds have medium mutagenic potential, low tumorigenic potential, high irritant potential and low reproductive effectivity.

### 2.4. Antineoplastic and Anticarcinogenic Activity Results

The predicted antineoplastic and anticarcinogenic activity for the screened compounds is given in Table 2 (PASS server). All compounds were predicted to have high antineoplastic activity (Pa > 0.5). The comparative anticarcinogenic analysis of the Pa and Pi values showed that **1**, **2** and **4** had a Pa value above 0.3. Compounds are classified as potentially bioactive if they exhibit a Pa value over the 0.3 threshold on the basis of precedents [26].

### 2.5. Density functional theory Calculation Results

**Figure 2:**
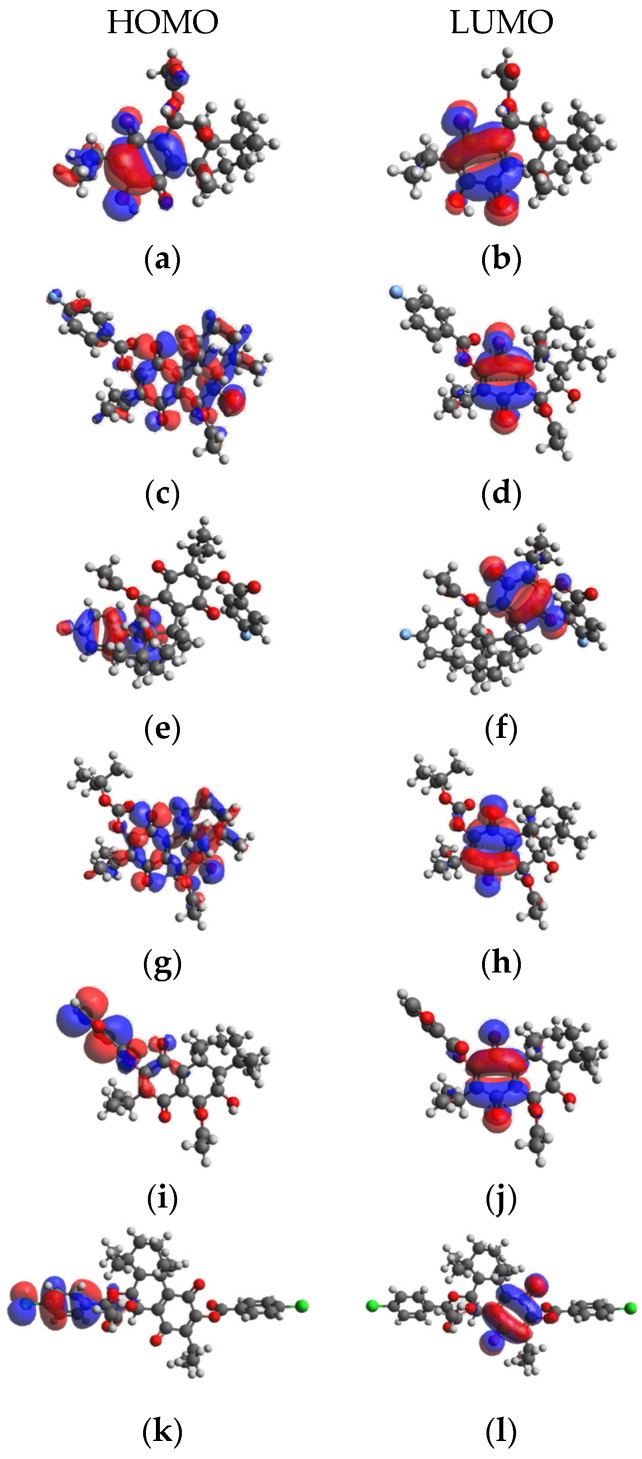
HOMO-LUMO diagram of compounds **1**–**6**. (**a**) HOMO energy of compound **1**; (**b**) LUMO energy of compound **1**; (**c**) HOMO energy of compound **2**; (**d**) LUMO energy of compound **2**; (**e**) HOMO energy of compound **3**; (**f**) LUMO energy of compound **3**; (**g**) HOMO energy of compound **4**; (**h**) LUMO energy of compound **4**; (**i**) HOMO energy of compound **5**; (**j**) LUMO energy of compound **5**; (**k**) HOMO energy of compound **6**; (**l**) LUMO energy of compound **6**.

The frontier molecular orbitals the high occupied molecular orbitals (HOMO) energies and low unoccupied molecular orbital (LUMO) provide valuable data on reactivity and stability. The density functional theory (DFT)-optimized conformations of compounds **1**–**6** are presented in Figure 2.

The HOMO and LUMO energies and relevant global reactivity descriptors are presented in Table 3. The LUMO energy refers to the electron-accepting aptitude of a molecule while the HOMO energy determines its electron-donating ability. In addition, ΔN is an important index, widely recognized to help predict the chemical reactivity and stability of inhibitors. The difference in HOMO and LUMO energies plays a significant role in comprehending chemical reactivity and kinetic stability. For example, a smaller HOMO–LUMO gap indicates a more polarizable molecule with lower kinetic stability and higher chemical reactivity. The HOMO–LUMO gaps for the compounds **1**–**6** were determined to be 2.63 to 3.74 eV. The lowest gap was found for compound **3**, indicating the highest chemical reactivity; a smaller band gap indicates that less energy is needed for the molecule to get excited from the ground level. The widest gap was observed for compound **4**, making it the least chemically reactive. 

The quantum chemical parameters hardness (η), softness (S), electronegativity (χ) and electrophilicity (ω) are global descriptors for the chemical behavior of molecules. The hardness value determines how an atom resists charge transfer to another atom or metal surface, while the softness value indicates the ability of an atom to receive electrons. Electronegativity (χ) is a chemical property that describes a molecule’s tendency to attract electrons, and the electrophilicity index (ω) determines the electrophilic property of a molecule [27]. Compound **3** was found to have lower hardness and higher softness, while **2** has a highest electronegativity index and **3** has the highest electrophilicity index.

### 2.6. Molecular Docking Results

**Figure 3:**
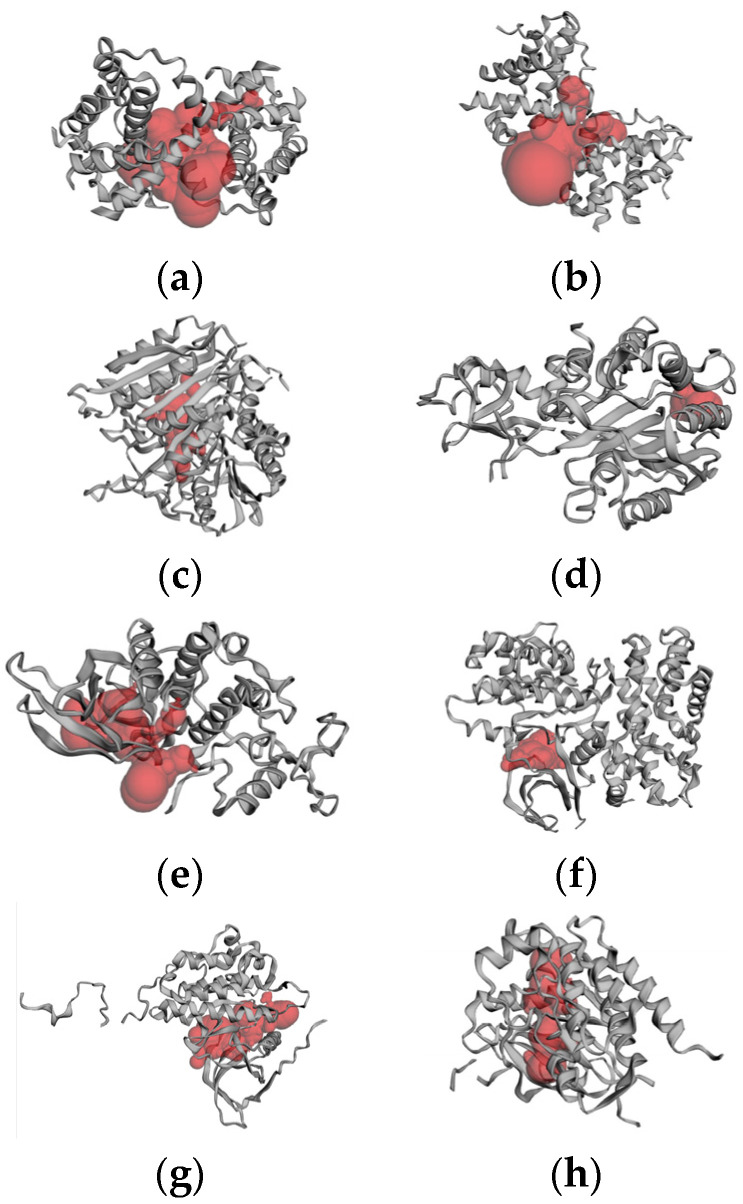

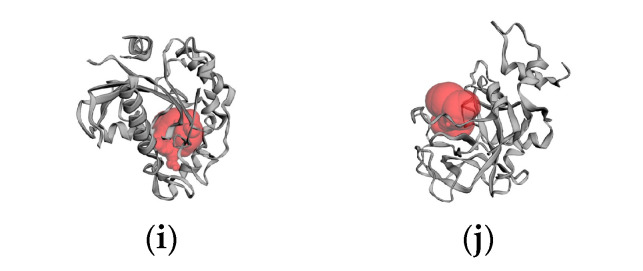
CAST-p pocket estimation: (**a**) BCL-2; (**b**) BCL-XL; (**c**) caspase 3; (**d**) caspase 9; (**e**) CDK2; (**f**) CDK6; (**g**) EGFR; (**h**) VEGFR; (**i**) p53; (**j**) PARP-1.

The protein structures were subjected to CAST-p analysis, followed by molecular docking. The ideal pocket areas (SA) for the targets BCL-2, BCL-XL, caspase 3, caspase 9, CDK2, CDK6, EGFR, VEGFR, p53 and PARP-1 were estimated to be 1097.807 Å, 1253.272 Å, 438.406 Å, 148.835 Å, 580.293 Å, 591.390 Å, 306.524 Å, 437.826 Å, 380.470 Å and 317.230 Å. The predicted active site regions of the target proteins are given in Figure 3.

Molecular docking studies were conducted to examine the binding interactions between 6 selected compounds (**1**–**6**) and the active sites of 10 target proteins implicated in cancer-related pathways. The proteins included key apoptosis, cell cycle, and growth factor signaling regulators associated with cancer pathogenesis, such as the anti-apoptotic proteins BCL-2 and BCL-XL [28], apoptotic effector caspases 3 and 9 [29], cell cycle promoting CDKs 2 and 6, growth factor receptors EGFR and VEGFR [30], tumor suppressor p53 [31], and the DNA repair protein PARP-1 [32]. These 10 cancer-related proteins represent crucial nodes across signaling cascades driving cell proliferation, survival, angiogenesis and genetic stability in malignant cells. Investigating the interactions between these proteins and the selected compounds using structure-based methods could aid in the identification of potential anticancer agents with selective activity against high-priority targets.

The docking results yielded predicted ligand–protein binding energies (kcal/mol) for the compounds bound to particular target proteins. Specifically, compound **5** showed strong predicted affinity for target proteins BCL-2 (complex 1), CDK2 (complex 2), EGFR (complex 3) and VEGFR (complex 4), with binding energies of −10.90 kcal/mol, −13.42 kcal/mol, −10.12 kcal/mol and −12.29, respectively. Meanwhile, compound **3** exhibited binding energies of −10.28 kcal/mol, −10.89 kcal/mol, −16.00 kcal/mol and −13.58 kcal/mol for target proteins caspase 3 (complex 5), CDK6 (complex 6) and PARP-1 (complex 7); compound **6** exhibited binding energies of −12.28 kcal/mol, −11.31 kcal/mol and −11.58 kcal/mol for targets proteins BCL-XL (complex 8), caspase 9 (complex 9) and p53 (complex 10). A more negative binding energy indicates more favorable compound–target binding potential. 

To validate the docking method, known inhibitors were also docked to the proteins BCL-2, BCL-XL, caspase 3, CDK2, CDK6, EGFR, VEGFR and PARP-1. The inhibitors used were BCL-2 inhibitor 1-[2-[(3S)-3-(aminomethyl)-3,4-dihydro-1H-isoquinoline-2-carbonyl]phenyl]-4-chloro-5-methyl-N,N-diphenylpyrazole-3-carboxamide (−9.11 kcal/mol), BCL-XL inhibitor N-[3-[5-[(E)-N-(1,3-benzothiazol-2-ylamino)-C-methylcarbonimidoyl]furan-2-yl]phenyl]sulfonyl-6-phenylhexanamide (−14.04 kcal/mol), caspase 3 inhibitor 5-[[4-[[(2S)-1-carboxy-3-oxobutan-2-yl]carbamoyl]phenyl]methylsulfamoyl]-2-hydroxybenzoic acid (−10.58 kcal/mol), CDK2 inhibitor 3-[(6,7-dimethoxyquinazolin-4-yl)amino]phenol (−9.72 kcal/mol), CDK6 inhibitor 2-(3,4-dihydroxyphenyl)-3,7-dihydroxychromen-4-one (−10.86 kcal/mol), EGFR inhibitor N-(3-ethynylphenyl)-6,7-bis(2-methoxyethoxy)quinazolin-4-aminE (−8.87 kcal/mol), VEGFR inhibitor methyl N-[6-[4-[[2-fluoro-5-(trifluoromethyl)phenyl]carbamoylamino]phenoxy]-1H-benzimidazol-2-yl]carbamate (−15.33 kcal/mol) and PARP-1 inhibitor 4-[[3-[4-(cyclopropanecarbonyl)piperazine-1-carbonyl]-4-fluorophenyl]methyl]-2H-phthalazin-1-one (−13.71 kcal/mol). The binding energies of these known inhibitors provide a reference point for assessing the predicted affinities of the studied compounds, compounds 1–6.

**Figure 4:**
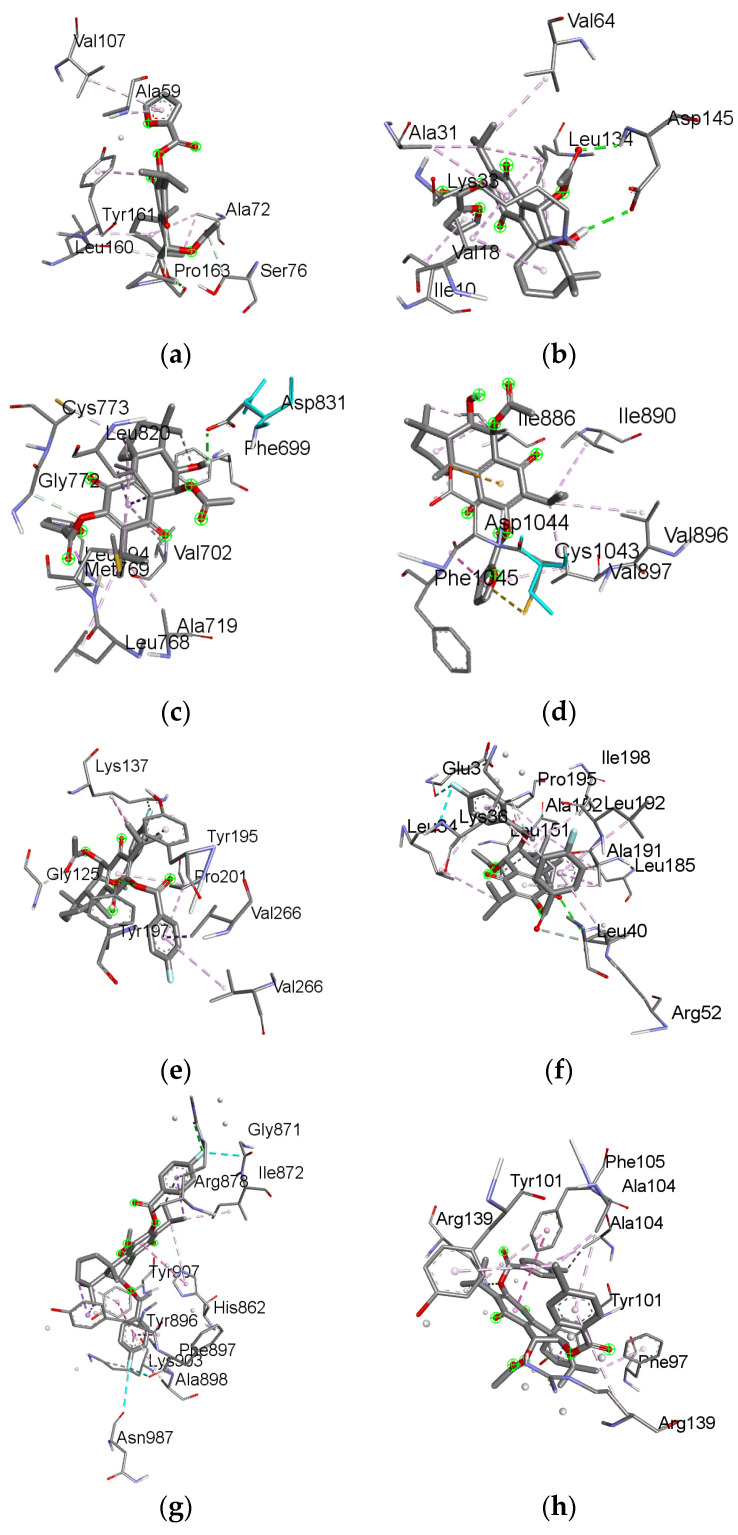

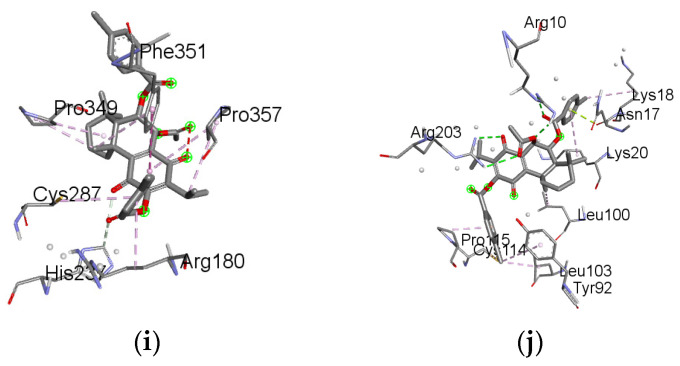
The 3D interactions of the **5** ligand against BCL-2 (**a**), CDK2 (**b**), EGFR (**c**) and VEGFR (**d**); **3** ligand against caspase 3 (**e**), CDK6 (**f**) and PARP-1 (**g**); **6** ligand against BCL-XL (**h**), caspase 9 (**i**) and p53 (**j**).

Further analysis of the docking poses revealed key molecular interactions underpinning the strong predicted affinities of **5**, **3** and **6** for relevant targets (Supplementary Table S11). The binding modes and ligand–protein interactions of **5**, **3** and **6** are depicted in Figure 4. Overall, the promising docking results for compounds **5** (BCL-2, CDK2, EGFR and VEGFR), **3** (caspase 3, CDK6 and PARP-1) and **6** (BCL-XL, caspase 9 and p53) suggest that these may be potent inhibitors against the therapeutically relevant proteins.

### 2.7. Molecular Dynamics Simulation Results 

The dynamic aspects of the docking complexes are simulated for 100 ns. The three top compounds were 5, which complexed with target proteins BCL-2 (complex 1), CDK2 (complex 2), EGFR (complex 3) and VEGFR (complex 4); 3, which complexed with target proteins caspase 3 (complex 5), CDK6 (complex 6) and PARP-1 (complex 7); 6, which complexed with target proteins BCL-XL (complex 8), caspase 9 (complex 9) and p53 (complex 10). MDSs were applied to investigate the stability of the proteins and ligands during their interaction based on the root mean square deviation (RMSD), the root mean square fluctuation (RMSF), the radius of gyration (Rg) and the solvent accessible surface area (SASA). 

**Figure 5:**
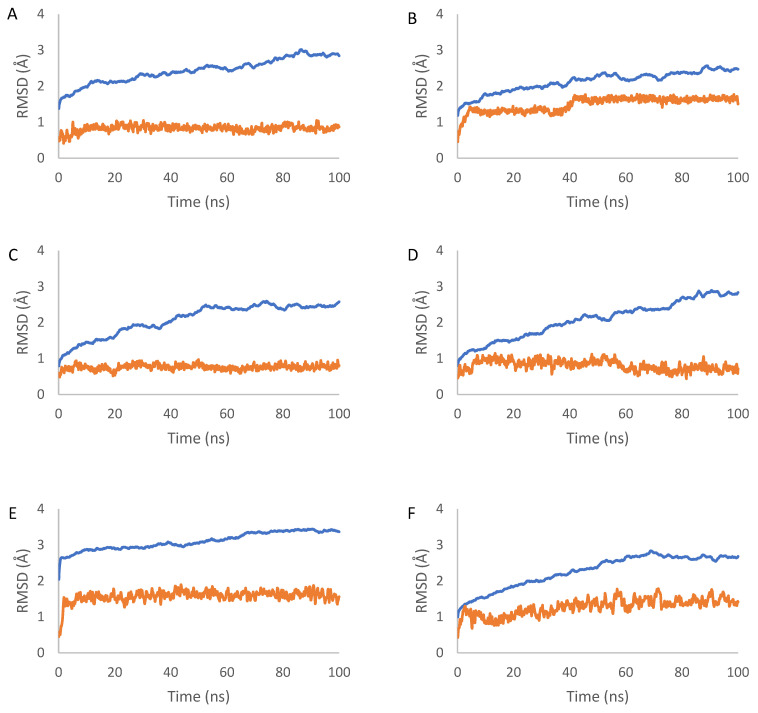

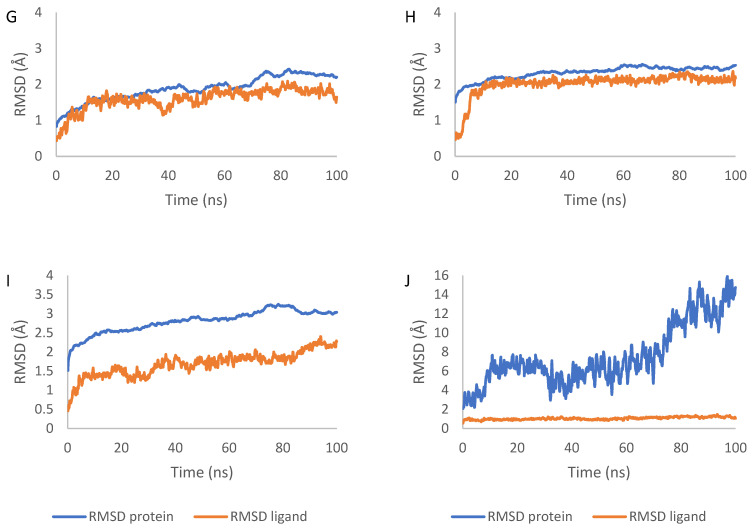
The RMSD values of the protein backbone and ligand atoms in the 100 ns MD simulation time for each of the 10 complexes. The RMSD plots, labeled (**A**–**J**), correspond to complexes 1–10, respectively. In each plot, the blue line represents the RMSD values for the protein backbone atoms, while the orange line represents the RMSD values for the ligand atoms.

RMSD was used to measure the average displacement of atoms in the protein and ligand relative to their initial positions, providing an assessment of their respective structural stabilities and conformational changes during the simulations. The RMSD values of the proteins backbone plateaued around 2.5 Å to 3.4 Å after approximately 20 to 60 ns, depending on the protein. The RMSD values of the proteins ranged from 0.14 nm to 0.30 nm for complex 1 (mean 0.24 nm), between 0.12 nm and 0.26 nm for complex 2 (mean 0.21 nm), between 0.08 nm and 0.26 nm for complex 3 (mean 0.21 nm), between 0.08 nm and 0.29 nm for complex 4 (mean 0.21 nm), between 0.20 nm and 0.34 nm for complex 5 (mean 0.31 nm), between 0.01 nm and 0.28 nm for complex 6 (mean 0.23 nm), between 0.08 nm and 0.24 nm for complex 7 (mean 0.19 nm), between 0.15 nm and 0.26 nm for complex 8 (mean 0.23 nm), between 0.15 nm and 0.32 nm for complex 9 (mean 0.28 nm) and between 0.21 nm and 1.6 nm for complex 10 (mean 0.75 nm). Structural mean fluctuations were less than 0.3 nm, suggesting that the complex attained a stable equilibrium, but the p53 complex with compound 6 showed some inconsistent higher fluctuations (Figure 5).

The high fluctuations observed in the p53 protein during the MD simulation of its complex with compound 6 suggest less stable conformation compared with that of p53 in other complexes. This instability may be attributed to the specific structural features of the p53 binding pocket and its inherent flexibility. The inconsistent fluctuations could indicate that the p53 protein undergoes significant conformational changes when compound 6 is bound, potentially affecting the stability of the complex. This dynamic behavior of p53 may influence the binding of compound 6 and the overall stability of the complex over time. The structural flexibility of p53 should be considered when interpreting the docking results and the potential of compound 6 as a p53 inhibitor, as it may impact the compound’s ability to form stable and persistent interactions with the protein. The ligands’ RMSD increased at the start of the simulation and then remained relatively stable at around 40 ns, with very low variation until the end of the simulation. The RMSD values of the ligands ranged between 0.04 nm and 0.10 nm for complex 1 (mean 0.08 nm), between 0.05 nm and 0.18 nm for complex 2 (mean 0.15 nm), between 0.05 nm and 0.01 nm for complex 3 (mean 0.08 nm), between 0.04 nm and 0.11 nm for complex 4 (mean 0.08 nm), between 0.05 nm and 0.19 nm for complex 5 (mean 0.16 nm), between 0.04 nm and 0.17 nm for complex 6 (mean 0.13 nm), between 0.04 nm and 0.21 nm for complex 7 (mean 0.16 nm), between 0.05 nm and 0.24 nm for complex 8 (mean 0.20 nm), between 0.05 nm and 0.24 nm for complex 9 (mean 0.17 nm) and between 0.05 nm and 0.15 nm for complex 10 (mean 0.10). These mean values, of less than 0.15 nm, indicate the stability of the docking interaction poses (Figure 5).

The 100 ns MD simulations showed that the ligands remained stably bound within the binding pocket of the protein over the course of the trajectory, indicating the formation of a stable protein–ligand complexes.

**Figure 6:**
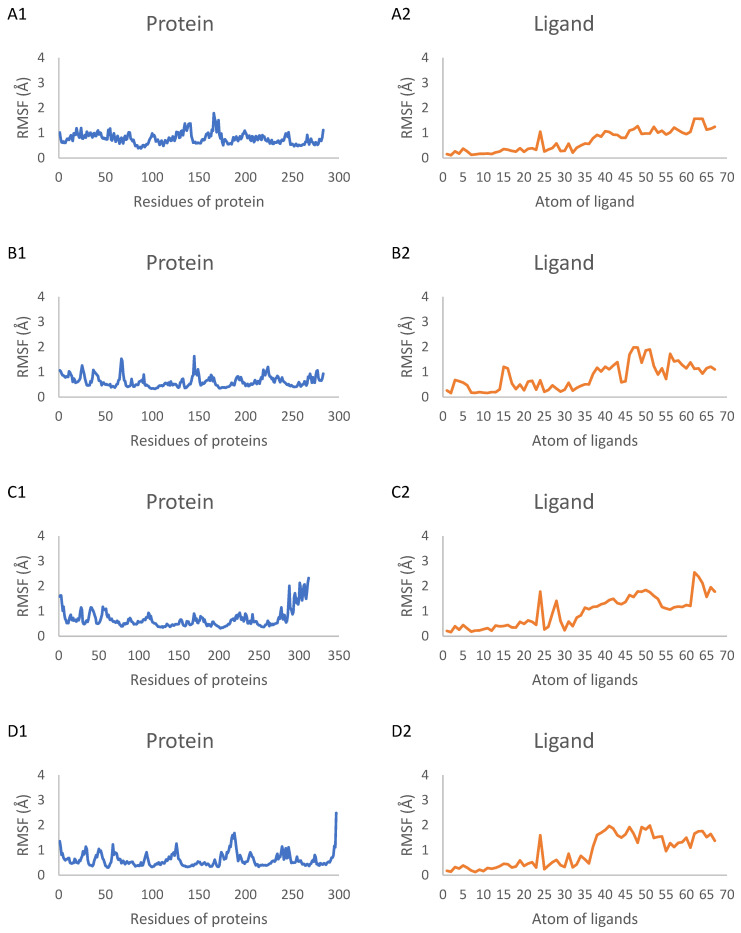

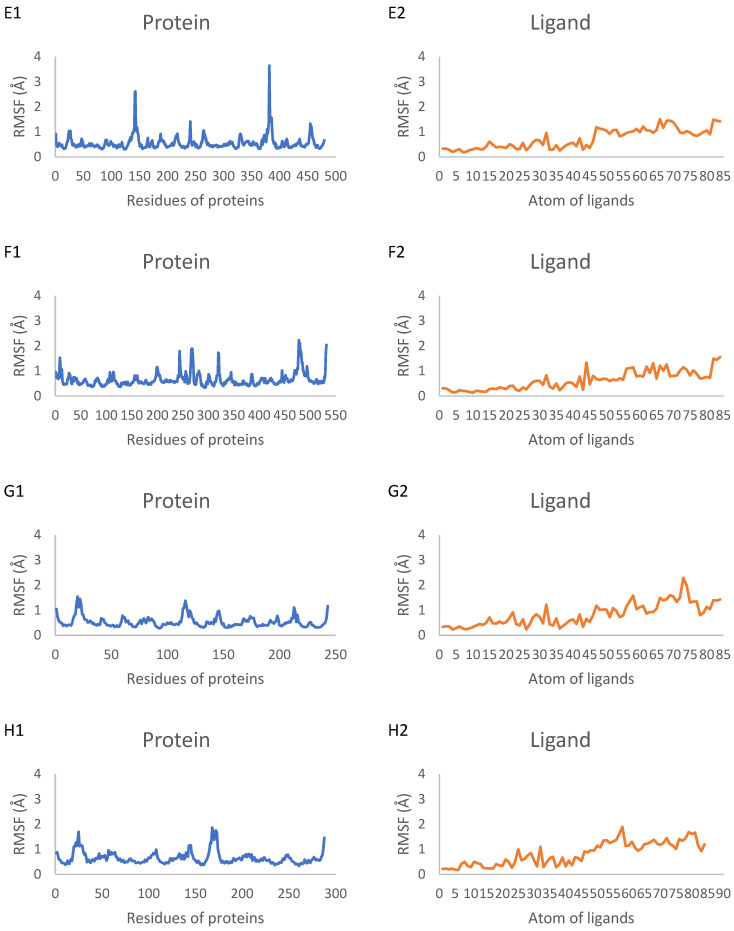

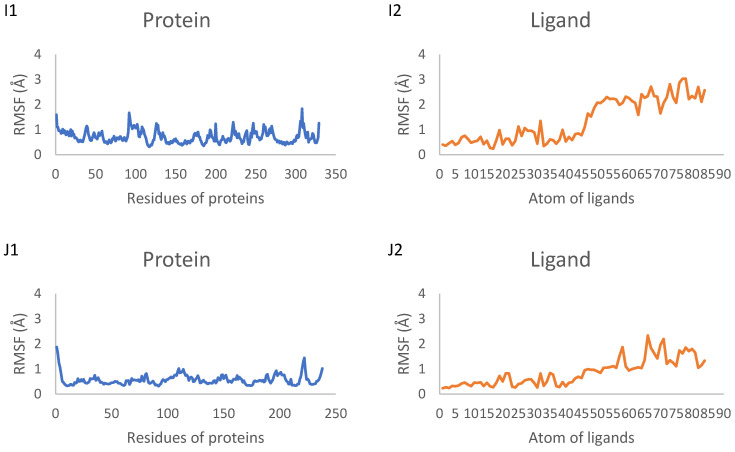
RMSF values for proteins and ligands in complexes 1–10. (**A1**–**J1**) RMSF values for backbone atoms of proteins in complexes 1–10, respectively, represented by blue lines. (**A2**–**J2**) RMSF values for all atoms of ligands in complexes 1–10, respectively, represented by orange lines.

The RMSF of individual protein residues and ligand atoms was computed to assess local flexibility and identify regions of high mobility in each component. The RMSF values were calculated for the backbone atoms of the proteins in complexes 1–10. As depicted in Figure 6, the RMSF values for most protein residues were consistently below 0.2 nm, indicating a high level of structural stability throughout the simulations. However, some exceptions were observed: the proteins in complexes 3, 4 and 6 exhibited maximum RMSF values of 0.32 nm, 0.25 nm and 0.22 nm, respectively. Additionally, caspase 3 complexed with compound 3 showed the highest fluctuation, with a maximum RMSF value of 0.36 nm. Despite these exceptions, the overall low RMSF values across the protein structures suggest that the proteins maintained a relatively rigid conformation, with limited fluctuations in their backbone positions. This observation provides evidence for the general stability of the complexes and the absence of significant conformational changes that could potentially disrupt the protein–ligand interactions. The RMSF values for all atoms of the ligands in complexes 1–10 were also calculated and are presented in Figure 6. Among the ligands, compound 6 complexed with caspase 9 exhibited the highest fluctuations, indicating that compound 6 underwent a more significant dynamic shift at the binding site compared with the other ligands in their respective complexes.

**Figure 7:**
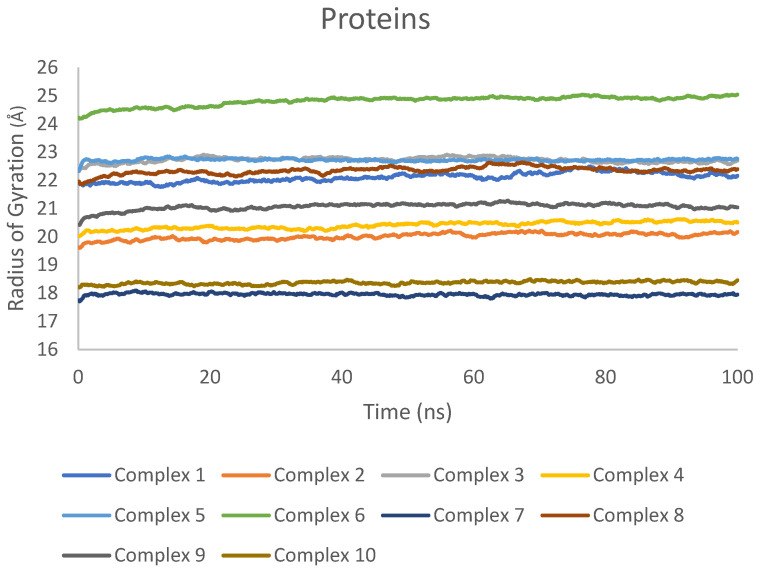
Rg values for proteins in complexes 1–10 during t100 ns MD simulations.

Rg was determined separately for the proteins and ligands to evaluate their compactness and overall dimensions throughout the simulations. The Rg values for the proteins in complexes 1–10 remained relatively stable, with only minor fluctuations observed (Figure 7). This indicates that the proteins maintained their overall structural integrity and did not undergo significant conformational changes during the MD simulations. 

**Figure 8:**
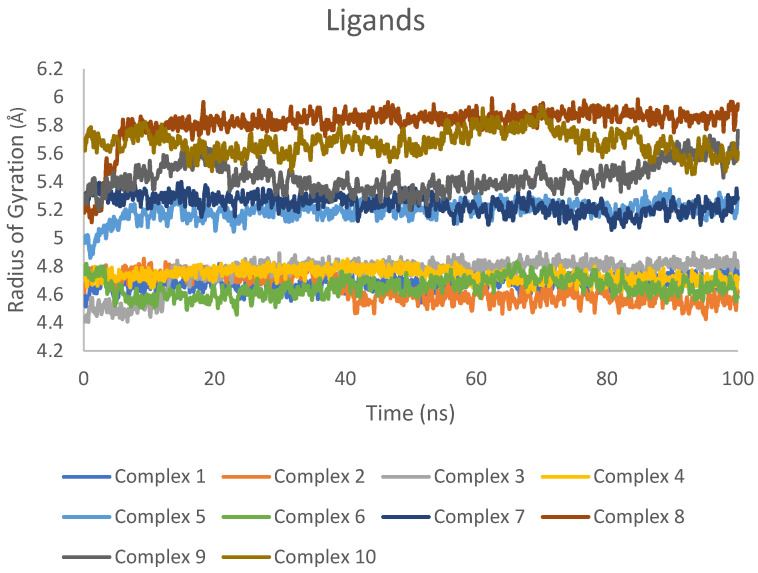
Rg values for ligands in complexes 1–10 during 100 ns MD simulations.

Similarly, the Rg values for the ligands in complexes 1–10 exhibited minimal variations (Figure 8), suggesting that the ligands maintained a stable conformation within the binding site. 

The consistency of Rg values for both proteins and ligands across all investigated complexes demonstrates the overall stability of the systems during the MD simulations. Moreover, the analysis of Rg also indicates that the proteins and ligands did not experience major conformational changes, further supporting the structural stability of the complexes.

**Figure 9:**
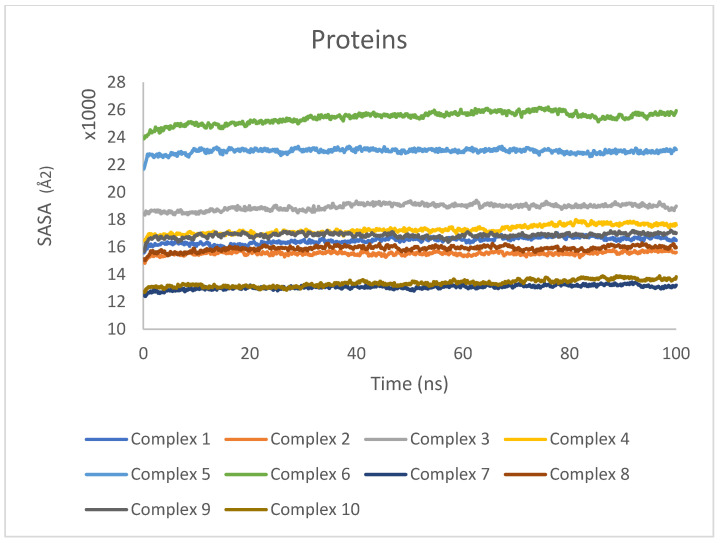
SASA values for proteins in complexes 1–10 during 100 ns MD simulations.

**Figure 10:**
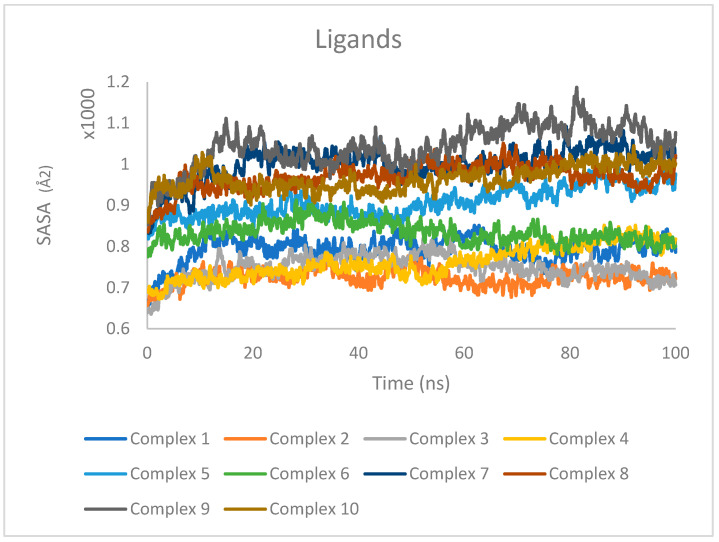
SASA values for ligands in complexes 1–10 during 100 ns MD simulations.

SASA was calculated individually for the protein and ligand to quantify the extent of their exposure to the surrounding solvent environment. The SASA values for the proteins are presented in Figure 9, while the SASA values for the ligands are shown in Figure 10.

For the proteins (Figure 9), the SASA values exhibited minor fluctuations but remained relatively stable overall, indicating that the proteins maintained a consistent level of solvent exposure throughout the simulations. These results suggest that the proteins did not undergo significant conformational changes that would drastically alter their solvent accessibility.

In the case of the ligands (Figure 10), the SASA values were found to be consistent over the entire trajectory of the simulation, with only minor fluctuations observed. The consistency in SASA values suggests that the ligands maintained a stable binding mode within the protein binding sites, without undergoing significant changes in their solvent accessibility. The minor fluctuations in SASA values can be attributed to the dynamic nature of the ligand–protein interactions and the flexibility of the ligands within the binding pockets.

### 2.8. Network Pharmacology Results 

**Figure 11:**
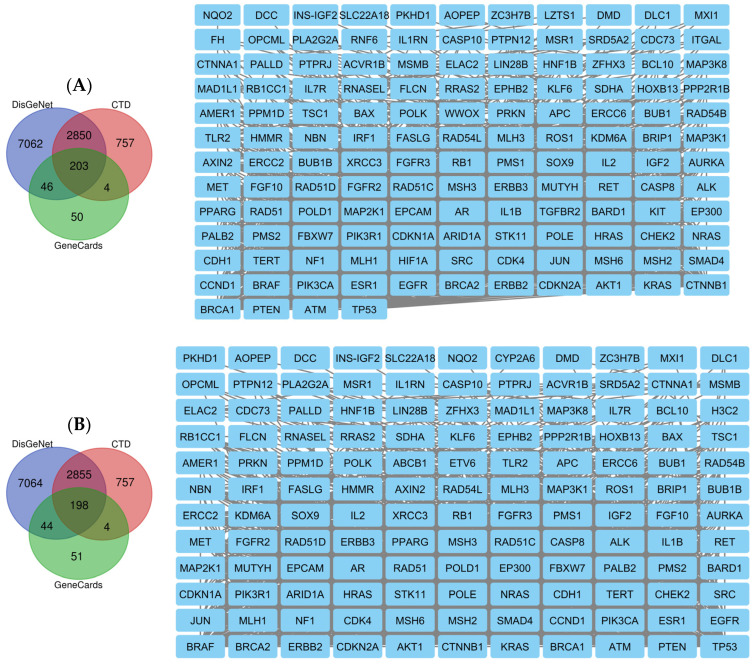

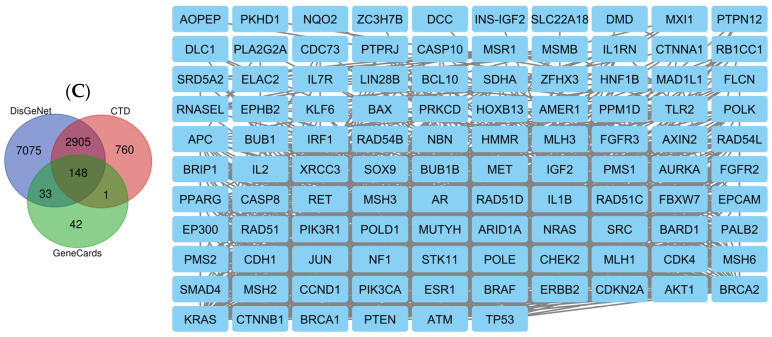
Compound **1** (**A**)-, compound **2**–**5** (**B**)- and compound **6** (**C**)-associated targets of cancers, zoomed in to show the specific targets.

Androgen receptor (AR), hypoxia-inducible factor 1-alpha (HIF1A) and integrin alpha-L (ITGAL) are the top-ranking targets for compound **1** identified via BindingDB, DrugBank, ChEMBL and SwissTargetPrediction. Meanwhile, the main target for compounds **2**–**5** is P-glycoprotein 1 (ABCB1) and that for compound **6** is the protein kinase C delta type (PRKCD). Using the keywords ‘neoplasms and cancer’, 10,161 potential neoplasm targets were retrieved from the DisGeNet database, 3814 neoplasm targets were retrieved from the CTD database for all compounds and 305, 298 and 225 cancer targets were retrieved from the GeneCards database from compounds **1**, **2**–**5** and **6**, respectively. In total, 203, 199 and 148 cancer targets were generated from the intersection of these three databases based on a Venn diagram for compounds **1**, **2**–**5** and **6**, respectively (Figure 11).

Proteins interact with each other to participate in diverse biological processes such as biological signaling, the regulation of gene expression, energy and material metabolism, and cell cycle regulation. In this study, protein-protein interaction networks (PPI) analysis between the compounds and associated cancers targets was performed to elucidate the underlying mechanisms using STRING. 

**Figure 12:**
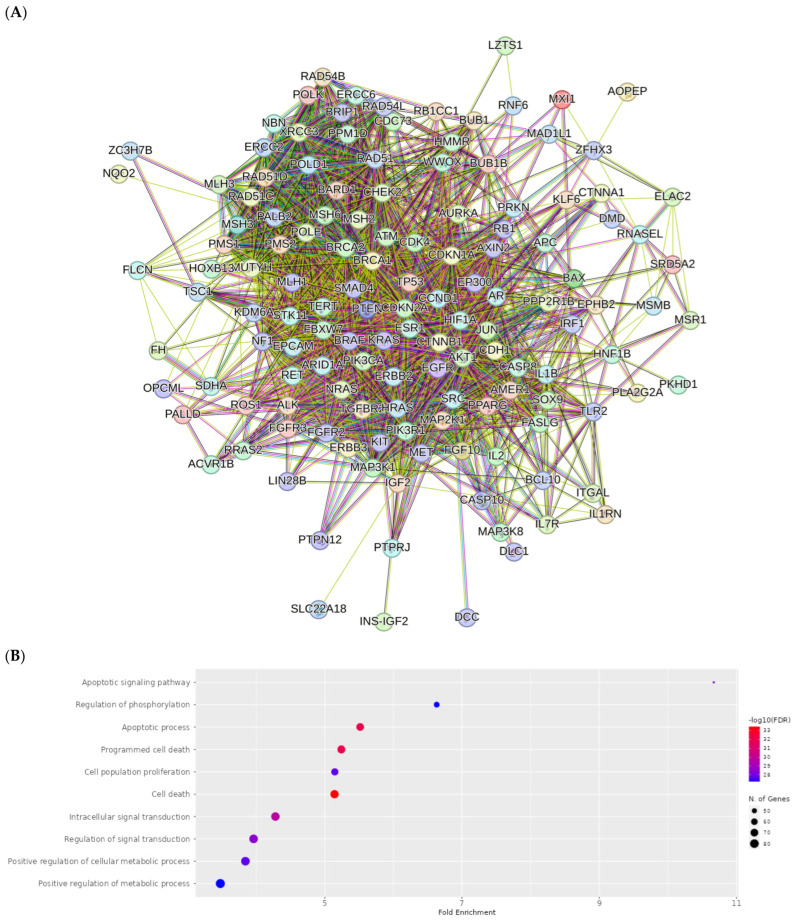

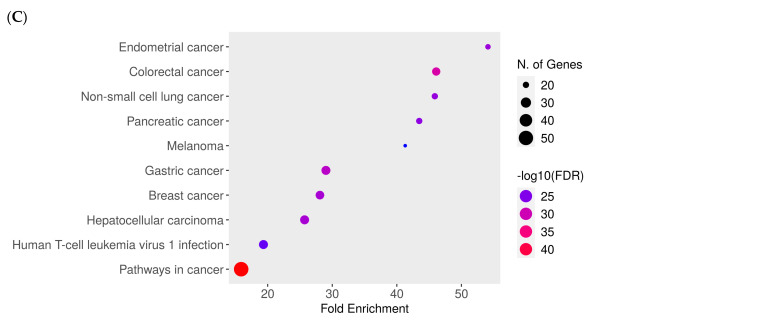
PPI network of 203 compound **1**-associated targets of cancers (**A**); GO analysis of all targets (**B**); KEGG analysis of all targets (**C**).

The PPI network for compound **1** is visualized in Figure 12A. Importantly, *TP53, ATM, PTEN, CTNNB1, BRCA1, KRAS, AKT1, CDKN2A, BRCA2* and *ERBB2* were identified as core compound **1** targets in cancer. The GO analysis of target genes of cancer targets was related to the regulation of the apoptotic signaling pathway, and many others (Figure 12B). According to KEGG analysis, most of the target genes were involved in pathways in cancer (Figure 12C).

**Figure 13:**
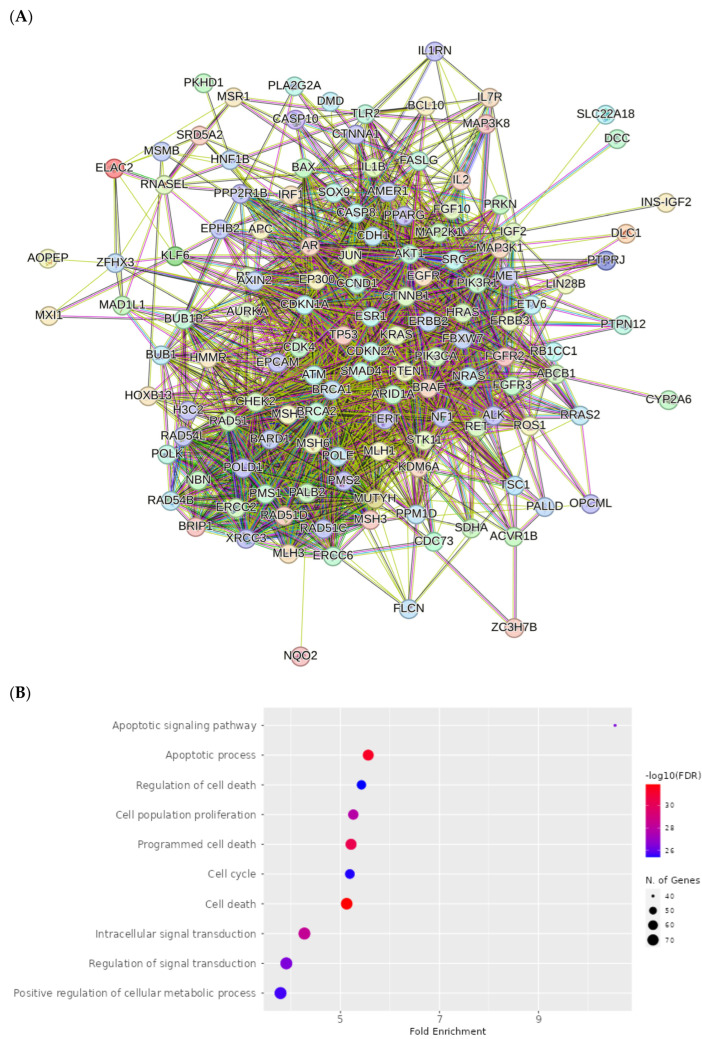

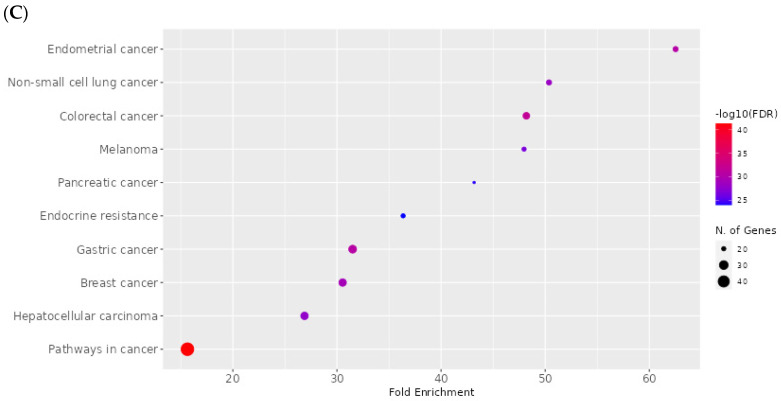
PPI network of 199 compound **2**–**5**-associated targets of cancers (**A**); GO analysis of all targets (**B**); KEGG analysis of all targets (**C**).

The PPI network for compounds **2**–**5** is visualized in Figure 13A. Importantly, *TP53*, *PTEN*, *ATM*, *BRCA1*, *KRAS*, *CTNNB1*, *AKT*, *CDKN2A*, *ERBB2* and *BRCA2* were identified as core compound 2–5 targets in cancer. The GO analysis of target genes of cancer targets was related to the regulation of the apoptotic signaling pathway, and many others (Figure 13B). According to KEGG analysis, most of the target genes were involved in pathways in cancer (Figure 13C).

**Figure 14:**
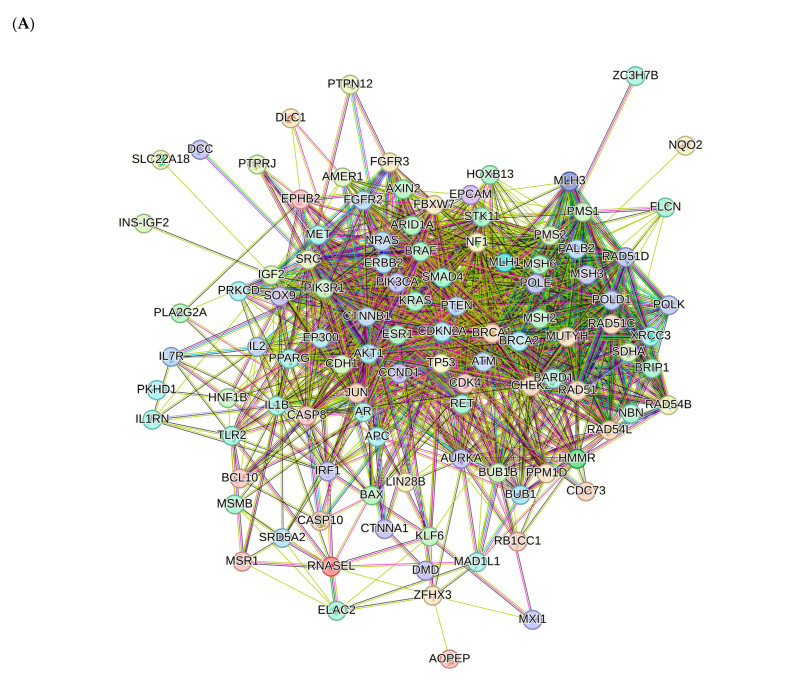

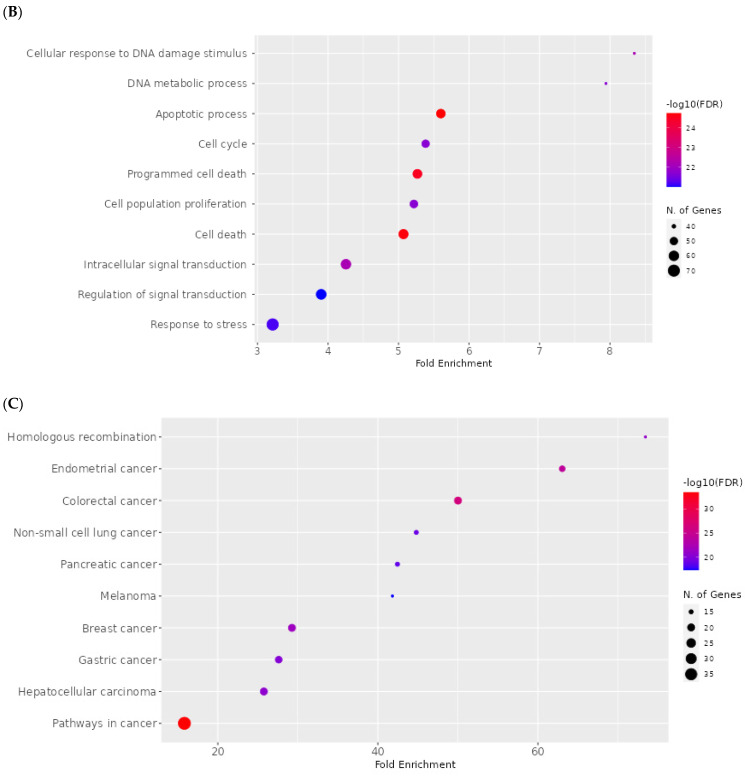
PPI network of 148 compound **6**-associated targets of cancers (**A**); GO analysis of all targets (**B**); KEGG analysis of all targets (**C**).

The PPI network for compound **6** is visualized in Figure 14A. Importantly, *TP53*, *ATM*, *PTEN*, *BRCA1*, *KRAS*, *CTNNBB1*, *BRCA2*, *AKT1*, *ERBB2* and *CDKN2A* were identified as core compound **6** targets in cancer. The GO analysis of target genes of cancer targets was related to the regulation of the response to stress, and many others (Figure 14B). According to KEGG analysis, most of the target genes were involved in pathways in cancer (Figure 14C).

## 3. Discussion

Plants constitute a valuable source of biologically active compounds, providing both essential nutrients and metabolites beneficial for maintaining human health and treating diseases [33,34]. Although plant-derived bioactive compounds are known to be beneficial, there remains an ongoing need to discover novel molecules with therapeutic potential [35]. In silico rational design based on predictive modeling is an important strategy as it can allow strategic chemical modifications of plant secondary metabolites through semisynthesis, which may further enhance or attenuate their biological activities, and serve as a viable starting point for biological assays and in vivo investigations [36].

The abietane diterpenoids isolated from *Plectranthus* species exhibit diverse bioactivities with therapeutic potential [37,38]. In particular, 7α-acetoxy-6β-hydroxyroyleanone (compound 1—Roy), a compound with antibacterial, anti-inflammatory and cytotoxic properties, has emerged as a promising candidate for five semisynthesis efforts focused on enhancing bioactivity via structural derivatization and preliminary assessment via computational modeling. 

Roy displays strong antibacterial activity against methicillin-resistant *Staphylococcus aureus* strains with a minimum inhibitory concentration (MIC) of 62.5 μg/mL [39]. Due to its high antibacterial potency and low cytotoxicity, Roy is a promising alternative to current antibiotics for treating infections caused by resistant bacteria. Roy interacts with bacterial cell membranes but does not significantly affect their permeability. Treated bacteria display cell wall disruption, without observable cell lysis; this suggests an as-yet undescribed mechanism of action that requires further investigation. It is possible that the compound penetrates into the cell interior and then induces alterations in the bacterial cell wall [40].

Roy also displays potent anti-inflammatory activity through the inhibition of the enzyme 5-lipoxygenase (5-LO), which is a key mediator of inflammation, mediating it via leukotriene biosynthesis. Roy was found to suppress the activity of isolated human recombinant 5-LO in cell-free assays with an IC_50_ of 1.3 μg/mL. Additionally, it effectively blocked 5-LO product formation in human neutrophils stimulated with calcium ionophore plus arachidonic acid, with an IC_50_ of 5.1 μg/mL [39].

Roy demonstrates cytotoxicity against various cancer cell lines including breast cancer (MCF7, SkBr3, SUM159 and MDA-MB-231S) [41,42], multiple glioblastoma (U87, A172, U118 and U373) [43] and lung cancer cells (NCI-H460 and NCI-H460/R) [44]. It shows potent antiproliferative effects across multiple cancer cell lines with low IC_50_ values in the micro-molar range. Its cytotoxic effects may be attributed to the activation of pro-apoptotic signaling cascades via protein kinase C (PKC) isozymes. Apoptosis or programmed cell death is crucial for regulating tissue development and homeostasis. Evasion of apoptosis is a hallmark of cancer, and its induction in malignant cells is a key therapeutic strategy [45]. Specifically, the PKC-δ and PKC-ζ isoforms have recognized roles in promoting apoptosis [46]. PKC-δ phosphorylates pro-apoptotic proteins like p53, Bax and caspase-3, leading to the implementation of the cell death program [47,48,49]. Meanwhile, PKC-ζ activates the JNK pathway and increases the expression of pro-apoptotic genes [50]. Roy may also trigger downstream signaling events, leading to apoptosis induction in cancer cells, as indicated by its potential to activate multiple PKC isozymes including PKC-δ and PKC-ζ. Moreover, even subtle structural changes in royleanones can greatly impact selectivity towards individual PKC isoforms [41].

The conducted in silico studies found five semisynthetic derivatives of the natural diterpenoid 7α-acetoxy-6β-hydroxyroyleanone (Roy) to have promising anticancer potential. 

The predicted ADMET parameters and toxicity profiles showed that the investigated compounds demonstrate desirable pharmacokinetic properties and selectivity, with a moderate risk of side effects. Derivative **3** had the lowest HOMO–LUMO energy gap, indicating high kinetic stability and low chemical reactivity. Molecular docking simulations to a panel of 10 cancer-associated protein targets found derivative **5** to have high affinity to BCL-2, CDK2, EGFR and VEGFR proteins, while derivative **3** exhibited favorable interactions with caspases 3, CDK6 and PARP-1; these compounds hence appear to be promising candidates as inhibitors of the proteins, which are therapeutically relevant apoptotic, cell cycle and growth factor regulators. The dynamic aspects of the top-ranking protein–ligand complexes were further examined using 100 ns molecular dynamics simulations. An analysis of the RMSD trajectories revealed the stable equilibration of the protein backbones and the conservation of key binding interactions over the course of the simulations. For the compound **5**–Bcl2, **5**–CDK2, **5**–EGFR and **5**–VEGFR complexes, protein RMSD values plateaued between 0.14 and 0.30 nm, while ligand RMSD values remained under 0.10 nm. Similarly, stable trajectories were observed for the compound **3**–caspase 3, **3**–CDK6 and **3**–PARP-1 systems, with mean protein and ligand RMSD ranges of 0.20–0.34 nm and 0.04–0.21 nm. This conservation of the docking poses in the dynamic binding sites supports the predicted strong affinities and indicates the potential for the selective inhibition of the protein targets.

Structure–activity relationship analysis revealed the significant impact of the introduced substituents on the predicted properties and the anticancer activity of the 7α-acetoxy-6β-hydroxyroyleanone derivatives. Compounds with single-ester substituents at the C-12 position (2, 4, 5) exhibited more favorable ADMET profiles and lower toxicity compared with the derivatives with double substituents at both the C-6 and C-12 positions (3, 6). The introduction of fluorine-containing aromatic substituents (2, 3 and 5) improved the affinity towards target proteins involved in cell proliferation and angiogenesis, such as EGFR and VEGFR, while aliphatic substituents (4) showed a preference for anti-apoptotic proteins like BCL-2 and BCL-XL.

The calculated DFT descriptors provided valuable insights into the electronic properties and reactivity of the investigated compounds. Compound 3 exhibited the lowest HOMO–LUMO energy gap (2.63 eV) and the highest global electrophilicity index (9.18 eV), indicating its higher chemical reactivity and potential for stronger interactions with biological targets. The low HOMO–LUMO gap suggests that compound 3 may be more easily polarizable and prone to electron transfer reactions, which could contribute to its binding affinity and biological activity. In contrast, compound 4, with the highest HOMO–LUMO gap (3.74 eV), is expected to be more chemically stable and less reactive.

Interestingly, a correlation was observed between the DFT descriptors and the predicted activity and ADMET properties of the compounds. Derivatives with lower HOMO–LUMO gaps and higher global electrophilicity indices (3, 5 and 6) generally showed better docking scores and binding affinities towards the selected protein targets. These compounds also demonstrated more favorable pharmacokinetic profiles, including higher gastrointestinal absorption and blood–brain barrier permeability. On the other hand, compounds with higher HOMO–LUMO gaps and lower electrophilicity indices (1, 2 and 4) exhibited relatively weaker binding affinities and less-than-optimal ADMET properties. Additionally, the network pharmacology approaches provided useful insights into the potential mechanisms of action of the compounds. The construction of protein–protein interaction networks revealed crucial nodes across signaling cascades related to apoptosis, cell proliferation, angiogenesis and DNA repair. Enrichment analysis indicated the involvement of cancer-associated pathways. Importantly, the integration of target prediction and network analysis facilitated the mapping of key biological processes and pathways that could be affected by the compounds to exert their anticancer effects.

Our multi-faceted in silico data of these Roy derivatives will be invaluable in future structure optimization efforts focused on enhancing their anticancer efficacy and drug-likeness. Together with the obtained docking energetics and predicted toxicity profiles, the encouraging molecular dynamics findings indicate that derivatives **5** and **3** are promising candidates for further experimental evaluation.

## 4. Materials and Methods

### 4.1. Compounds

#### 4.1.1. General Experimental Procedures

Nuclear magnetic resonance (NMR) spectra were recorded on a Bruker Fourier 300 spectrometer and a Bruker Fourier 400 spectrometer (Bruker, Billerica, MA, USA). Melting points were determined on a Stuart Scientific SMP10 AC/DC input, 230 V (Merck, Darmstadt, Germany). Optical rotations were measured on an Anton Paar MC100 polarimeter (in CHCl3 solution) (Anton Paar, Graz, Austria). Attenuated total reflectance Fourier-transform infrared spectroscopy (ATR-FT-IR) spectra were collected on a Perkin-Elmer Spectrum Two FT-IR spectrophotometer with Universal ATR (Perkin-Elmer, Shelton, CT, USA). LC-HRMS was performed on a Dionex Ultimate 3000 UHPLC+ system equipped with a multiple-wavelength detector, using an imChem Surf C18 TriF 100 A 3 µm 100 × 2.1 mm column, a linear solvent gradient from 20 to 30% aqueous ACN over 10 min, 0.2 mL of min^−1^, and UV detection at 250 nm, connected to a Thermo Scientific Q-Exactive hybrid quadrupole-Orbitrap mass spectrometer (Thermo Scientific, Waltham, MA, USA). All solvents were distilled from commercial-grade sources. Pyridine (Merck, Darmstadt, Germany) and all benzoyl and anhydride reagents were used without any previous purification.

#### 4.1.2. Plant Material

The plant material *P. grandidentatus* Gürke was cultivated in Parque Botânico da Tapada da Ajuda (Instituto Superior Agrário, Lisbon, Portugal) from cuttings obtained from the Kirstenbosch National Botanical Garden (Cape Town, South Africa). Voucher specimens were deposited in Herbarium João de Carvalho e Vasconcellos (ISA) with the number 841/2007. The plant material used in this study was collected between 2007 and 2008, and then dried and stored at room temperature, protected from light and humidity. The plant name Was checked using The Plant List and World Flora Online databases (http://www.theplantlist.org and http://www.worldfloraonline.org/, accessed on 8 September 2023).

#### 4.1.3. Extraction and Isolation

Acetone ultrasonic-assisted extraction was adapted from Bernardes et al. [51]. The dry aerial parts of *P. grandidentatus* were immersed in acetone and subjected to three 30 min extractions in a Sonorex Super RK 510 H ultrasonic bath (Bandelin, Berlin, Germany) at room temperature (35 Hz, maximum input power 320 W). The solvent was then filtered and evaporated under vacuum at 40 °C; the process resulted in an extraction yield of 2.3% *w*/*w*. The crude extract was subjected to sequential chromatographic separations. Finally, substance **1** was obtained as yellow crystal plates, via recrystallization from n-Hexane. The spectral data are in agreement with those in the literature [43,51,52,53].

The general esterification procedure was performed in accordance with the optimized conditions reported in Isca et al. [54]. 

The selection of carboxylic acid derivatives for the esterification of 7α-acetoxy-6β-hydroxyroyleanone was based on the concept of halogen substitution to potentially enhance the biological activity of the parent compound. Fluorine and chlorine substituents were introduced to modulate the physicochemical properties and pharmacological profile of the resulting esters, as halogenated compounds have a proven track record in drug discovery. The specific derivatives (4-fluorobenzoyl chloride, 2-furoyl chloride, pivaloyl chloride and 4-chlorobenzoyl chloride) were chosen to cover a range of structural features, including aromatic and aliphatic moieties with varying degrees of bulkiness and halogen substitution patterns. This approach aimed to obtain a focused library of 7α-acetoxy-6β-hydroxyroyleanone esters for preliminary structure–activity relationship studies [55,56,57,58].

The esterification reactions of 7α-acetoxy-6β-hydroxyroyleanone (1) with various carboxylic acid derivatives yielded products with different substitution patterns. Compounds 2 4, and 5 were obtained through a single esterification reaction at the 12-OH position, while compounds 3 and 6 underwent esterification at both the 6-OH and 12-OH positions.

The selectivity of the esterification reactions can be attributed to the differences in the reactivity and accessibility of the two hydroxyl groups in the parent compound. The 12-OH group is less sterically hindered and more accessible compared with the 6-OH group, which is located in a more congested region of the molecule. This difference in a steric environment likely favors the preferential esterification of the 12-OH group under the applied reaction conditions.

In the case of compounds 3 and 6, the use of a larger excess of the carboxylic acid derivative (4-fluorobenzoyl chloride and 4-chlorobenzoyl chloride, respectively) and/or prolonged reaction times likely contributed to double esterification. The increased concentration of the acylating agent and longer exposure may have allowed for the esterification of the more sterically hindered 6-OH group after the initial reaction at the 12-OH position [59,60].

**Figure 15:**
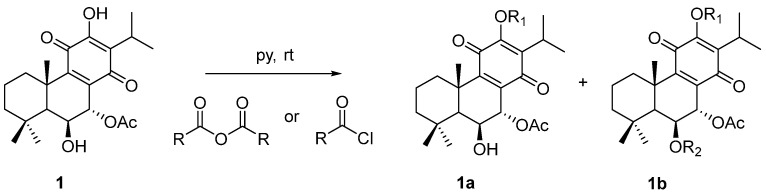
Reaction of semisynthesis of Roy **1** ester derivatives.

To a solution of **1** (20 μmol, 1 equiv.) in pyridine (0.5 mL, under inert conditions), an excess of the corresponding benzoyl chloride or acetic anhydride (3–21 equiv.) was added. The reaction was stirred and allowed to sit at room temperature, under inert conditions, until the full conversion of **1**. The crude mixture was purified via preparative chromatography (Figure 15). 

The NMR and MS results of compounds 7α-acetoxy-6β-hydroxyroyleanone (**1**), 7α-acetoxy-6β-hydroxy-12-*O*-(4-fluoro)benzoylroyleanone (**2**), 7α-acetoxy-6β-(4-fluoro)benzoxy-12-*O*-(4-fluoro)benzoylroyleanone (**3**), 7α-acetoxy-6β-hydroxy-12-*O*-trimethylacetylroyleanone (**4**) and 7α-acetoxy-6β-hydroxy-12-*O*-(2-fluoryl)royleanone (**5**) are presented in the Supplementary Materials. The synthesis of compound 7αacetoxy-6β-benzoyloxy-12-*O*-(4-chloro)benzoylroyleanone (**6**) was presented previously [61].

### 4.2. ADMET and Drug-Likeness Analysis

ADMET (absorption, distribution, metabolism, excretion and toxicity) and drug-likeness properties were predicted using the SwissADME server (http://www.swissadme.ch/, accessed on 2 October 2023). Physicochemical parameters including molecular weight (MW), the count of specific atom types, molecular refractivity (MR) and polar surface area (PSA) were determined. Lipophilicity was assessed using iLOGP, XLOGP3, WLOGP, MLOGP and SILICOS-IT models. These models were utilized to establish a consensus log P_octanol/water (log Po/w) value. Water solubility (log S) was predicted using three different models: ESOL, Ali and SILICOS-IT. Pharmacokinetic parameters such as GI absorption, BBB permeability, P-gp substrate potential, CYP1A2 inhibition, CYP2C19 inhibition, CYP2C9 inhibition, CYP2D6 inhibition, CYP3A4 inhibition and Log Kp (skin permeation) were determined. Rule-based filters, including Lipinski, Ghose, Veber, Egan and Muegge methods were employed to estimate the drug-likeness of molecules. Medicinal chemistry descriptors were predicted [62,63].

### 4.3. Toxicity Prediction and Molecular Properties

The compounds were subjected to toxicity analysis. The ProTox-II server (http://tox.charite.de/protox_II, accessed on 4 October 2023) provided the LD50 values in mg/kg body weight and toxicity statuses, especially hepatotoxicity, carcinogenicity, immunotoxicity, mutagenicity, cytotoxicity and signaling, for the following nuclear receptors: AhR, AR, AR-LBD, ER, ER-LBD and PPAR-Gamma. It also examined the following stress response pathways: nrf2/ARE, HSE, MMP, phosphoprotein p53 and ATAD5 [64]. The StopTox server (https://stoptox.mml.unc.edu/, accessed on 5 October 2023) was employed to determine the acute toxicity associated with exposure to the compounds [65]. The OSIRIS server (https://www.organic-chemistry.org/prog/peo/, accessed on 6 October 2023) was used to determine the probability of the compounds being irritants, mutagenic agents or tumorigenic agents, and their effect on reproduction [66].

### 4.4. Anticarcinogenic Activity

The potential anticarcinogenic properties in selected compounds were assessed using PASS (prediction of activity spectra for substances). PASS is an application (http://www.pharmaexpert.ru/passonline/, accessed on 6 October 2023) used to forecast diverse activities across various molecules. The predictive spectrum generated via PASS relies on the analysis of structural activity relationships (SAR) within the training dataset. The projected activity spectrum of a compound is presented as probable activity (Pa) or probable inactivity (Pi). Compounds with higher Pa values in comparison with those of Pi are deemed plausible candidates for a particular activity [67].

### 4.5. DFT Calculations

The ligand’s structure was created in Avogadro v1.95 and saved in xyz format. The DFT calculations were carried out in the gas phase using the Orca v5.03 package and the B3LYP/def2-TZVPP basis set [68]. The DFT results were analyzed using Avogadro [69].

### 4.6. Molecular Docking

#### 4.6.1. Protein and Ligand Preparation

The three-dimensional structures of BCL-2 (PDB ID: 2W3L), BCL-XL (PDB ID:3ZK6), caspase 3 (PDB ID: 1NMS), caspase 9 (PDB ID: 1NW9), CDK2 (PDB ID: 1DI8), CDK6 (PDB ID: 1XO2), EGFR (PDB ID: 1M17), VEGFR (PDB ID: 2OH4), p53 (PDB ID: 3DCY) and PARP1 (PDB ID: 5DS3) were retrieved from the PDB database. Protein receptors were saved as.pdb files. Discovery Studio Visualizer was used to delete heteroatoms. AutoDock Tools v1.5.6 was used to eliminate water molecules from the basic structure. Missing atoms were repaired. Polar hydrogens and Kollman charges were added. The resulting optimized .xyz coordinates for the ligands were then used in molecular docking [70].

#### 4.6.2. Active Site Prediction

The active site location for all screened compounds was determined using Computed Atlas Surface Topography of Proteins—CAST-p (http://sts.bioe.uic.edu/castp/index.html?3trg, accessed on 6 November 2023). This a particular web server was used to predict the volume, area and configuration of active sites dedicated to ligand binding [71].

#### 4.6.3. Receptor–Ligand Docking

Molecular docking studies were performed using AutoDock software v1.5.7 [72]. Based on the results of CAST-p, grid boxes were set according to the location of active sites. The positions of the grid boxes, BCL-2 (X = 34.530, Y = 31.076, Z = 1.645), BCL-XL (X = 19.061, Y = 51.294, Z = 0.154), caspase 3 (X = 6.758, Y = −0.081, Z = 9.017), caspase 9 (X = 53.800, Y = 18.030, Z = 92.471), CDK2 (X = −4.908, Y = 47.554, Z = 14.485), CDK6 (X = 29.145, Y = 43.770, Z = 131.559), EGFR (X = 30.025, Y = 4.113, Z = 52.110), VEGFR (X = 3.072, Y = 38.530, Z = 15.593), PARP-1 (X = −2.393, Y = 39.874, Z = 13.085), were selected with a 0.500 angstrom grid point spacing. All calculations were based on the Lamarckian genetic algorithm method. The analysis consisted of 100 independent runs, with a population size of 300. Individual docking procedures were conducted for each ligand–protein complex. The outcomes were organized in ascending order of docking energies, with the lowest binding energy from each cluster considered representative. Two-dimensional and three-dimensional interaction maps were created using BIOVIA Discovery Studio Visualizer [73]. To provide a reference for the docking scores and binding affinities of the investigated compounds, control ligands with known activity against protein targets were also docked. The control ligands were selected based on the co-crystallized inhibitors present in the PDB structures of the respective proteins. For p53 and caspase-9, no co-crystallized ligands were available in the selected PDB structures; therefore, no control ligands were used for these targets. The docking scores of the control ligands were compared with those of the investigated compounds to assess the relative binding strengths and potential for inhibitory activity.

### 4.7. Molecular Dynamics Simulations 

Molecular dynamics simulations (MDS) of selected protein–ligand complexes were performed using NAMD software v2.14 [74] with CHARMM36m force field parameters [75]. The topology files were generated using the CHARMM-GUI server [76]. The complexes were solvated in a rectangular water box with an edge distance of 10 Å. Additionally, the Monte Carlo method was used to add KCl ions at a concentration of 0.15 M. Energy minimization was performed using the steepest descent method (5000 steps). Two stages of equilibration for 100 ps were carried out to bring the system to a constant temperature (303.15 K) and pressure (1 atm). The first equilibration was performed in the NVT ensemble (constant number of particles, volume and temperature), while the second was performed in the NPT ensemble (constant number of particles, pressure and temperature). Finally, the MD simulation production step was conducted with a simulation time of 100 ns using an integration time of 2 fs. The VMD program [77] is used to retrieve the resulting simulation data, which include root mean square deviation (RMSD), root mean square fluctuation (RMSF), radius of gyration (Rg) and solvent-accessible surface area (SASA), and RMSD calculation used the coordinates after every 100 ps.

### 4.8. Network Pharmacology 

#### 4.8.1. Identification of Potential Targets of Analyzed Compounds

Potential targets of analyzed compounds were identified and collected using the BindingDB database (https://www.bindingdb.org/bind/index.jsp, accessed on 5 February 2024), DrugBank (https://go.drugbank.com/, accessed on 5 February 2024), ChEMBL (https://www.ebi.ac.uk/chembl/, accessed on 5 February 2024) and SwissTargetPrediction (http://www.swisstargetprediction.ch/, accessed on 5 February 2024).

#### 4.8.2. Associated Targets of Cancer Diseases

DisGeNet (https://www.disgenet.org/, accessed on 5 February 2024), the comparative toxicogenomics database, CTD (https://ctdbase.org/, accessed on 5 February 2024) and the human gene database, GeneCards (https://www.genecards.org/, accessed on 5 February 2024), were used to identify cancer targets with the keywords ‘neoplasms and cancer’. To identify connections between cancer target genes and active components, the online application Venny v2.1.0 (https://bioinfogp.cnb.csic.es/tools/venny/, accessed on 5 February 2024) was used.

#### 4.8.3. Visualization and Analysis of the Network of the Protein–Protein Interactions

In order to examine protein–protein interactions (PPI), the STRING platform (https://string-db.org/, accessed on 6 February 2024) was used to obtain human data sets with a confidence score exceeding 0.4. The resulting interaction network was then analyzed using Cytoscape v3.10.1 [78].

#### 4.8.4. Enrichment Analysis 

Biological process, molecular function and cellular component are three important parts of gene ontology (GO) (https://geneontology.org/docs/ontology-documentation/, accessed on 7 February 2024). GO and Kyoto Encyclopedia of Genes and Genomes (KEGG) (https://www.genome.jp/kegg/pathway.html, accessed on 7 February 2024) pathway analyses were conducted to predict the possible molecular mechanism of the analyzed compounds against cancer. Enrichment analyses were performed using ShinyGO v0.741 with an adjusted *p* value < 0.05. The top 10 terms identified were visualized in a diagram.

## 5. Conclusions

The integrated approach used in the study, combining molecular modeling, physicochemical and toxicological profiling, and quantum chemical calculations, enabled the efficient screening of a focused library of Roy derivatives. The findings constitute a solid premise for further in vitro tests of the most promising candidates (7α-acetoxy-6β-hydroxy-12-*O*-(2-fluoryl)royleanone (**5**) and 7α-acetoxy-6β-(4-fluoro)benzoxy-12-*O*-(4-fluoro)benzoylroyleanone (**3**)) to confirm their anticancer activity.

## Figures and Tables

**Table 1:** Predicted acute oral toxicity of the screened compounds obtained using the ProTox-II server.

Compounds	Predicted LD_50_ (mg/kg)	Predicted Toxicity Class	Prediction Accuracy (%)
**1**	1000	4	69.26
**2**	100	3	67.38
**3**	100	3	67.38
**4**	75	3	68.07
**5**	100	3	54.26
**6**	100	3	67.38

**Table 2:** Predicted antineoplastic and anticarcinogenic activity of the examined compounds obtained by using the PASS server.

Compounds	Antineoplastic Activity		Anticarcinogenic Activity	
	Pa Value	Pi Value	Pa Value	Pi Value
**1**	0.879	0.005	0.419	0.028
**2**	0.810	0.010	0.315	0.053
**3**	0.822	0.009	0.279	0.067
**4**	0.882	0.0005	0.312	0.054
**5**	0.770	0.016	0.255	0.080
**6**	0.811	0.010	0.289	0.063

**Table 3:** Frontier molecular orbitals, gap values and descriptors for the optimized structures of the compounds in the gas phase, obtained using the DFT method.

Compound	E_HOMO_ (eV)	E_LUMO_ (eV)	ΔE (eV)	I (eV)	A (eV)	χ (eV)	μ (eV)	η (eV)	S (eV^−1^)	ω (eV)	ΔN_max_
**1**	−6.885	−3.433	3.45	6.89	3.43	5.16	−5.16	1.73	0.29	7.71	2.99
**2**	−7.279	−3.572	3.71	7.28	3.57	5.43	−5.43	1.85	0.27	7.94	2.93
**3**	−6.229	−3.599	2.63	6.23	3.60	4.91	−4.91	1.32	0.38	9.18	3.74
**4**	−7.226	−3.488	3.74	7.23	3.49	5.36	−5.36	1.87	0.27	7.68	2.87
**5**	−7.081	−3.429	3.65	7.08	3.43	5.26	−5.26	1.83	0.27	7.56	2.88
**6**	−7.035	−3.493	3.54	7.04	3.49	5.26	−5.26	1.77	0.28	7.82	2.97

ΔE—ELUMO–EHOMO; I—ionization energy; A—electron affinity; χ—electronegativity; μ—chemical potential; η—global chemical hardness; S—global chemical softness; ω—global electrophilicity index; ΔN_max_—maximum additional electric charge.

## Data Availability

Data are contained within the article and Supplementary Materials.

## Supplementary Materials

The supporting information can be downloaded at https://www.mdpi.com/article/10.3390/ijms25084529/s1.
