# Supplementary material for: Exploring the Anticancer Potential of Semisynthetic Derivatives of 7α-Acetoxy-6β-hydroxyroyleanone from Plectranthus sp.: An In Silico Approach
Source: Int J Mol Sci. 2024 Apr 20;25(8):4529. doi: 10.3390/ijms25084529 (PMC11050557; doi:10.3390/ijms25084529)
Supplement: Supplementary file 1 [file ijms-25-04529-s001.zip › ijms-2924393-supplementary.pdf]

Supplementary materials

The FTIR, NMR and MS results of compounds: 7α-acetoxy-6β-hydroxyroyleanone (1), 7α-acetoxy-6β-hydroxy-12-*O*-(4-fluoro)benzoylroyleanone (2), 7α-acetoxy-6β-(4-fluoro)benzoxy-12-*O*-(4-fluoro)benzoylroyleanone (3), 7α-acetoxy-6β-hydroxy-12-*O*-trimethylacetylroyleanone (4), 7α-acetoxy-6β-hydroxy-12-*O*-(2-fluoryl)royleanone (5).

7α-acetoxy-6β-hydroxyroyleanone (1): ^{1}H-NMR (300 MHz, Chloroform-*d*, ppm): δ 7.22 (s, 1H, 12-OH), 5.66 (dd, J = 2.2, 0.7 Hz, 1H, H-7β), 4.31 (s, 1H, H-6α), 3.16 (sept, *J* = 7.1 Hz, 1H, H-15), 2.63 (d, *J* = 12.8 Hz, 1H, H-1β), 2.04 (s, 3H, 7α-OAc), 1.89– 1.78 (m, 1H, H-2β), 1.61 (s, 3H, Me-20), 1.55 – 1.46* (m, 2H, H-2α and H-3β), 1.33 (s, 1H, H-5α), 1.23* (s, 3H, Me-19), 1.22* (d, *J* = 7.1 Hz, 3H, Me-17), 1.21* (s, 1H, H-3α $^{+}$), 1.20* (d, *J* = 7.1 Hz, 3H Me-16), 1.18* (s,1H H-1α $^{+}$), 0.94 (s, 3H, Me-18). * overlapped signals, $^{+}$ can be changed.

7α-acetoxy-6β-hydroxy-12-O-(4-fluoro)benzoylroyleanone (2): The compound was prepared according to the general procedure, with 4-fluorobenzoyl chloride (929.0 µmol, 15 equiv.) then heated at 50°C overnight. The crude mixture was purified by preparative chromatography with mixture of hexane/ethyl acetate (8:2). The reaction afforded pure products **2** (44 %) and **3** (11 %). Derivative **2** was obtained as a yellow amorphous solid. **mp:** 192-194°C. $[\alpha]_D^{20} = +41.2$ ° (*c*, 0.340, $CHCl_3$). **IR $\bar{\nu}_{max}$:** 3528.5, 2969.9, 2929.4, 2868.7, 1751.5, 1666.5, 1605.8, 1512.7, 1468.2, 1371.0, 1253.7, 1217.3, 1140.4, 1108.0, 1063.5, 1027.0, 857.0, 759.9 cm^{-1}. **^{1}H NMR (400 MHz, Chloroform-*d*):** δ 8.17 (dt, *J* = 8.6, 5.4 Hz, 2H, H-2′ $^{+}$), 7.20 (t, *J* = 8.6 Hz, 2H, H-2′ $^{+}$), 5.68 (d, *J* = 2.0 Hz, 1H, H-7β), 4.34 (s, 1H, H-6α), 3.18 (qui, *J* = 7.1 Hz, 1H, H-15), 2.49 (s, 1H, H-1β), 2.07 (s, 3H, 7α-OAc), 1.89 – 1.73 (m, 1H, H-2β), 1.63 (s, 3H, Me-20), 1.55 (dt, *J* = 14.1, 3.7 Hz, 1H, H-2α), 1.46 (dt, *J* = 14.1, 3.3 Hz, 1H, H-3β), 1.37 (s, 1H, H-5α), 1.27 – 1.16* (m, 11H, Me-19, Me-17, H-3α, Me-16, H-1α), 0.95 (s, 3H, Me-18). * Overlapped signal; $^{+}$ can be changed. **^{13}C NMR (101 MHz, Chloroform-*d*):** δ 185.88 (C-14), 179.79 (C-11), 169.89 (7α-$\underline{C}$OCH$_3$), 167.94 (12-$\underline{C}$OO), 167.94 (C-1′ $^{+}$), 153.22 (C-9), 149.68 (C-12), 139.61 (C-13), 135.85 (C-8), 133.41 (C-3′)$^{++}$, 133.31 (C-3′ $^{++}$), 124.41 (C-4′ $^{+}$), 116.33 (C-2′ $^{++}$), 116.11 (C-2′)$^{++}$, 69.03 (C-7), 67.33 (C-6), 49.92 (C-5), 42.41 (C-3), 39.07 (C-10), 38.47 (C-1), 33.87 (C-4), 33.67 (C-18), 25.31 (C-15), 23.98 (C-19), 21.87 (C-20), 21.07 (7α-CO$\underline{C}$H$_3$), 20.59 (C-16), 20.37 (C-17), 19.02 (C-2). $^{+}$ and $^{++}$ can be changed. **HRMS (ESI-MS)** m/z calculated for $C_{29}H_{34}FO_7$ $[M+H]^+$ 513.2283, found 513.22858.

7α-acetoxy-6β-(4-fluoro)benzoxy-12-O-(4-fluoro)benzoylroyleanone (3): The pure derivative was obtained as yellow needles (11 %). **mp:** 162-164°C. $[\alpha]_D^{20} = -91.9$ ° (*c* 0.272, $CHCl_3$). **IR $\bar{\nu}_{max}$:** 2971.5, 2932.4, 2877.0, 1749.0, 1726.3, 1667.6, 1605.7, 1507.9, 1432.9, 1260.1, 1217.7, 1155.8, 1136.2, 1107.0, 1090.6, 1061.3, 1012.4, 852.6, 767.9, 738.5, 705.9, 685.4 cm^{-1}. **^{1}H NMR (300 MHz, Chloroform-*d*):** δ 8.18 (dd, *J* = 8.9, 5.3 Hz, 1H, H-3′ $^{+}$), 8.13 (dd, *J* = 9.0, 5.4 Hz, 1H, H-3′′ $^{+}$), 7.25 – 7.04 (m, 4H, H-2′, H-2′′), 5.89 (d, *J* = 1.7 Hz, 1H, H-7β), 5.78 (s, 1H, H-6α), 3.18 (p, *J* = 7.0 Hz, 1H, H-15), 2.61 (br s, 1H, H-1β), 2.11 (s, 3H, 7α-OAc), 1.87 – 1.77 (m, 1H, H-2β), 1.74 (d, *J* = 15.2 Hz, 3H, Me-20), 1.70 (s, 1H, H-5α), 1.66 – 1.55 (m, 1H, H-2α), 1.49 (dt, *J* = 13.5, 3.5 Hz, 1H, H-3β), 1.34* (dd, *J* = 5.2, 2.7 Hz, 1H, H-3α $^{++}$), 1.22 (dd, *J* = 7.0, 3.1 Hz, 7H, Me-16, Me-17, H-1α $^{++}$), 1.11 (s, 3H, Me-18), 0.99 (s, 3H, Me-19). * Overlapped signal; $^{+}$ and $^{++}$ can be changed. **^{13}C NMR (101 MHz, Chloroform-*d*)**: δ 185.43 (C-14), 168.29 (6-$\underline{C}$OO $^{+}$), 167.75 (C-1′′ $^{++}$), 164.54 (12-$\underline{C}$OO $^{+}$), 163.19 (C-1′ $^{++}$), 133.44 (C-3′ $^{+++}$), 133.31 (C-3 ′$^{+++}$), 133.08 (C-3′′ $^{++}$), 132.95 (C-3′′ $^{++}$), 126.16 (C-4′ $^{++}$), 124.36 (C-4′′ $^{++}$), 116.44 (C-2′ $^{+++}$), 116.15 (C-2′ $^{+++}$), 116.03 (C-2′′ $^{+++}$), 115.74 (C-2′′ $^{+++}$), 68.78 (C-6), 65.42 (C-7), 49.48 (C-5), 42.65 (C-3), 38.95 (C-10), 38.31 (C-1), 33.93 (C-4), 33.46 (C-18), 23.36 (C-15), 22.84 (C-19), 22.39 (C-20), 20.96 (7α-CO$\underline{C}$H$_3$), 20.56 (C-16), 20.50(C-17), 18.99 (C-2). $^{+}$, $^{++}$ and $^{+++}$ can be changed. **HRMS (ESI-MS):** m/z calculated for $C_{36}H_{36}F_2O_8$ $[M+Na]^+$ 657.22719, found 657.2270.

7α-acetoxy-6β-hydroxy-12-O-trimethylacetylroyleanone (4): The compound was prepared according to the general procedure, with pivaloyl chloride (408.7 µmol, 21 equiv.) then let to react for 50 min. The crude mixture was purified by preparative chromatography with mixture of dichloromethane/acetone (99:1). The pure product (87 %) was obtained as a yellow oil. $[\alpha]_D^{20} = +22.3$ ° (*c* 0.224, $CHCl_3$). **IR $\bar{\nu}_{max}$:** 3516.0, 2965.0, 2932.4, 2870.5,

1762.1, 1749.0, 1667.6, 1615.4, 1485.0, 1462.2, 1371.0, 1276.4, 1227.5, 745.1 cm^{-1}. **^{1}H NMR (300 MHz, Chloroform-*d*, ppm):** δ 5.65 (d, *J* = 1.9 Hz, 1H, H-7β), 4.32 (t, *J* = 1.7 Hz, 1H, H-6α), 3.12 (qui, *J* = 7.1 Hz, 1H, H-15), 2.49 (dt, *J* = 15.1, 5.6 Hz, 1H, H-1β), 2.05 (s, 3H, 7α-OAc), 1.80 (t, *J* = 13.5 Hz, 1H, H-2β), 1.63 (s, 3H, Me-20), 1.56 (dt, *J* = 14.2, 3.9 Hz, 1H, H-2α), 1.52 – 1.41 (m, 1H, H-3β), 1.38 (s, 9H, Me-2'), 1.33 (s, 1H, H-5α), 1.22 (s, 3H, Me-19), 1.19* (d, *J* = 7.1 Hz, 8H, Me-17, H-3α, Me-16, H-1α), 0.94 (s, 3H, Me-18). *Overlapped signal. **^{13}C NMR (75 MHz, Chloroform-*d*, ppm):** δ 185.82 (C-14 ⁺), 179.72 (C-11 ⁺), 171.76 (12-C̲OO), 169.73(7α-C̲OCH3), 154.27(C-9), 140.34(C-12), 139.33(C-13), 135.45 (C-8), 68.98 (C-7), 67.25 (C-6), 49.81(C-5), 42.13 (C-3), 39.23 (C-1'), 38.89(C-10), 38.19(C-1), 33.73(C-4), 33.58(C-18), 27.17 (C-2'), 25.16(C-15), 22.93(C-19), 21.75(C-20), 21.73(7α-COC̲H3), 21.27(C-16), 20.89(C-17), 18.91 (C-2). ⁺ Can be changed. **HRMS (ESI-MS)** m/z calculated for $C_{27}H_{38}O_7$ $[M+H]^+$ 475.26903, found 475.26853.

7α-acetoxy-6β-hydroxy-12-O-(2-fluoryl)royleanone (5): The compound was prepared according to the general procedure, with 2-furoyl chloride (230.5 μmol, 6 equiv.) then let to react for 10 min. The crude mixture was purified by preparative chromatography with dichloromethane. The pure product (49 %) was obtained as a yellow amorphous powder. **mp:** 190-192°C. $[\alpha]_D^{20}$ = + 55.7° (*c* 0.323, $CHCl_3$). **IR** $\bar{\nu}_{max}$**:** 3587.6, 2965.0, 2932.4, 2867.2, 1745.8, 1667.6, 1609.0, 1569.8, 1472.0, 1397.0, 1371.0, 1283.0, 1224.3, 1168.8, 1139.5, 777.7 cm^{-1}. **^{1}H NMR (300 MHz, Chloroform-*d*)**: δ 7.70 (dd, *J* = 1.8, 0.8 Hz, 1H, H-5' ⁺, 7.41 (d, *J* = 3.6, 0.8 Hz, 1H, H-3' ⁺), 6.61 (dd, *J* = 3.6, 1.8 Hz, 1H, H-4' ⁺), 5.68 (d, *J* = 1.7 Hz, 1H, H-7β), 4.34 (t, *J* = 1.7 Hz, 1H, H-6α) , 3.17 (qui, *J* = 7.1 Hz, 1H, H-15), 2.52 (br d, *J* = 12.8 Hz, 1H, H-1β), 2.06 (s, 3H, 7α-OAc), 1.87-1.73 (m, 1H, H-2β), 1.63 (s, 3H, Me-20), 1.56 (t, *J* = 3.7 Hz, 1H, H-2α), 1.53-1.47 (m, 1H, H-3β), 1.36 (s, 1H, H-5α), 1.24 – 1.20* (m, 11H, Me-19, Me-17, H-3α, Me-16, H-1α), 0.95 (s, 3H, Me-18). * Overlapped signal; ⁺ can be changed. **^{13}C NMR (75 MHz, Chloroform-*d*, ppm):** δ 185.72 (C-14), 179.51 (C-11), 169.69 (7α-C̲OCH3), 155.35 (12-C̲OO), 153.03 (C-9), 148.89 (C-12), 147.93 (C-5' ⁺), 142.75 (C-2'), 139.96 (C-13), 135.73 (C-8), 120.75 (C-3' ⁺), 112.46 (C-4' ⁺), 69.90 (C-7), 67.23 (C-6), 49.79 (C-5), 42.30 (C-3), 38.94 (C-10), 38.35 (C-1), 33.74 (C-4), 33.51 (C-18), 25.25 (C-15), 23.85 (C-19), 21.75 (C-20), 20.90 (7α-COC̲H3), 20.30 (C-16), 20.24 (C-17), 18.89 (C-2). **HRMS (ESI-MS):** m/z calculated for $C_{27}H_{32}O_8$ $[2M+Na]^+$ 991.4087, found 991.40850.

Supplementary Table S1. Predicted physicochemical properties of the screened compounds, obtained using SwissADME server.

Compounds	Formula	MW	No. heavy atoms	No. aromatic heavy atoms	Fraction Csp3	No. ratable bonds	No. H-bond acceptors	No. H-bond donors	Molar refractivity
1	C22H30O6	390.47	28	0	0.68	3	6	2	104.48
2	C29H33FO7	512.57	37	6	0.52	6	8	1	133.66
3	C36H36F2O8	634.66	46	12	0.42	9	10	0	163.26
4	C27H38O8	490.59	35	0	0.70	7	8	1	129.74
5	C27H32O8	484.54	35	5	0.56	6	8	1	125.97

6	C36H36Cl2O8	66.57	46	12	0.42	9	8	0	173.36

Supplementary Table S2. Predicted lipophilicity of the screened compounds obtained using SwissADME server.

Compounds	TPSA	iLOGP	XLOGP3	WLOGP	MLOGP	Silicos-IT LogP	Consensus LogP
1	100.90	3.11	3.36	3.04	1.43	3.22	2.83
2	106.97	3.99	5.23	4.90	3.19	5.28	4.52
3	113.04	4.95	7.56	7.32	4.84	7.31	6.40
4	116.20	4.37	4.86	4.44	2.02	4.22	3.98
5	120.11	3.90	4.53	3.93	1.68	4.21	3.65
6	113.04	5.05	8.62	7.51	5.02	7.75	6.79

Supplementary Table S3. Predicted water solubility of the screened compounds obtained using SwissADME server.

Compounds	ESOL log S	ESOL solubility [mg/mL]	ESOL solubility [mol/L]	ESOL class	Ali log S	Ali solubility [mg/mL]	Ali solubility [mol/L]	Ali class	Silicos-IT log S	Silicos-IT solubility [mg/mL]	Silicos-IT solubility [mol/L]	Silicos-IT class
1	-4.18	2.58e-02	6.61e-05	Moderately soluble	-5,16	2.72e-03	6.97e-06	Moderately soluble	-3.61	9.58e-02	2.45e-04	Soluble
2	-6.04	4.71e-04	9.19e-07	Poorly soluble	-7.22	3.05e-05	5.96e-08	Poorly soluble	-6.55	1.45e-04	2.83e-07	Poorly soluble
3	-8.14	4.63e-06	7.30e-09	Poorly soluble	-9.77	1.08e-07	1.70e-10	Poorly soluble	-9.45	2.24e-07	3.53e-10	Poorly soluble
4	-5.84	1.62e-03	3.30e-06	Moderately soluble	-7.03	4.53e-05	9.23e-08	Poorly soluble	-4.73	9.18e-03	1.87e-05	Moderately soluble

5	-5.41	1.89e-03	3.91e-06	Moderately soluble	-6.77	8.15e-05	1.68e-07	Poorly soluble	-5.51	1.48e-03	3.06e-06	Moderately soluble
6	-9.01	6.54e-07	9.80e-10	Poorly soluble	-10.87	9.01e-09	1.35e-11	Insoluble	-10.08	5.58e-08	8.36e-11	Insoluble

Supplementary Table S4. Predicted pharmacokinetics parameters of the screened compounds obtained using SwissADME server.

Compounds	GI adsorption	BBB permeant	Pgp substrate	CYP1A2 inhibitor	CYP2C19 inhibitor	CYP2C9 inhibitor	CYP2D6 inhibitor	CYP3A4 inhibitor	Log Kp [cm/s]
1	High	No	Yes	No	No	No	No	Yes	-6.30
2	High	No	Yes	No	No	Yes	No	Yes	-5.71
3	Low	No	Yes	No	No	Yes	No	No	-4.80
4	High	No	Yes	No	No	Yes	No	No	-5.84
5	High	No	Yes	No	No	Yes	No	Yes	-6.04
6	Low	No	Yes	No	No	Yes	No	No	-4.25

Supplementary Table S5. Predicted drug-likeness, medicinal chemistry and lead-likeness pharmacokinetics parameters of the screened compounds obtained using SwissADME server.

Compounds	Lipinski # violations	Ghose # violations	Veber # violations	Egan # violations	Muegge # violations	Bioavailability score	PAINS # alerts	Brenks # alerts	Leadlikeness # violations	Synthetic accessibility
1	0	0	0	0	0	0.56	1	1	1	5.23
2	1	2	0	0	1	0.56	1	2	2	5.65
3	2	4	0	1	2	0.56	1	2	3	6.16
4	0	2	0	0	0	0.56	1	2	2	5.09
5	0	1	0	0	0	0.56	1	2	2	5.73
6	2	4	0	1	2	0.56	1	2	3	6.15

Supplementary Table S6. Predicted organ toxicity and toxicological endpoints of the screened compounds obtained using ProTox-II server.

Compounds	Hepatotoxicity (% probability)	Carcinogenicity (% probability)	Immunotoxicity (% probability)	Mutagenicity (% probability)	Cytotoxicity (% probability)
1	Inactive (72)	Active (50)	Active (94)	Inactive (85)	Inactive (81)
2	Inactive (69)	Active (56)	Active (99)	Inactive (84)	Inactive (74)
3	Inactive (57)	Active (58)	Active (99)	Inactive (78)	Inactive (75)
4	Inactive (73)	Inactive (50)	Active (84)	Inactive (85)	Inactive (85)
5	Inactive (74)	Active (51)	Active (89)	Inactive (76)	Inactive (85)
6	Inactive (55)	Active (60)	Active (99)	Inactive (77)	Inactive (74)

Supplementary Table S7. Toxicological pathways: nuclear receptor signaling pathways predicted for the screened compounds obtained using ProTox-II server (% probability).

Compounds	Aryl Hydrocarbon Receptor (AhR)	Androgen Receptor (AR)	Androgen Receptor Ligand Binding Domain (AR-LBD)	Aromatase	Estrogen Receptor Alpha (ER)	Estrogen Receptor Ligand Binding Domain (ER-LBD)	Peroxisome Proliferator Activated Receptor Gamma (PPAR-Gamma)
1	Inactive (91)	Inactive (86)	Inactive (87)	Inactive (87)	Inactive (77)	Inactive (87)	Inactive (96)
2	Inactive (85)	Inactive (79)	Inactive (82)	Inactive (82)	Inactive (62)	Inactive (91)	Inactive (94)
3	Inactive 85)	Inactive (81)	Inactive (80)	Inactive (82)	Inactive (69)	Inactive (89)	Inactive (94)
4	Inactive (91)	Inactive (85)	Inactive (87)	Inactive (84)	Inactive (79)	Inactive (84)	Inactive (96)
5	Inactive (93)	Inactive (70)	Inactive (83)	Inactive (65)	Inactive (71)	Inactive (93)	Inactive (94)
6	Inactive (82)	Inactive (83)	Inactive (79)	Inactive (76)	Inactive (77)	Inactive (90)	Inactive (94)

Supplementary Table S8. Toxicological pathways: stress response pathways predicted for the screened compounds obtained using ProTox-II server (% probability).

Compounds	Nuclear Factor (Erythroid-Derived 2-Like 2/Antioxidant Responsive Element) (nrf2/ARE)	Heat Shock Factor Response Element (HSE)	Mitochondrial Membrane Potential (MMP)	Phosphoprotein (Tumor Suppressor) p53	ATPase Family AAA Domain Containing Protein 5 (ATAD5)
1	Inactive (76)	Inactive (76)	Inactive (58)	Inactive (93)	Inactive (84)
2	Inactive (91)	Inactive (91)	Inactive (51)	Inactive (82)	Inactive (86)
3	Inactive (84)	Inactive (84)	Active (52)	Inactive (70)	Inactive (87)
4	Inactive (84)	Inactive (84)	Inactive (58)	Inactive (93)	Inactive (86)
5	Inactive (92)	Inactive (92)	Active (50)	Inactive (87)	Inactive (85)
6	Inactive (84)	Inactive (84)	Active (56)	Inactive (57)	Inactive (85)

Supplementary Table S9. Predicted acute toxicity of the screened compounds obtained using StopTox server.

Compounds	Inhalation Toxicity	Oral Toxicity	Dermal Toxicity	Eye Irritation and Corrosion	Skin Sensitization	Skin Irritation and Corrosion
1	No	No	No	No	No	No
2	No	No	No	No	No	No
3	No	No	No	No	No	No
4	No	No	No	No	No	No
5	No	No	No	No	No	No
6	No	No	No	No	No	No

Supplementary Table S10. OSIRIS output of potential harmful properties of compounds

Compounds	Mutagenic Potential	Tumorigenic Potential	Irritant Potential	Reproductive Effectivity
1	Medium	Low	High	Low
2	Medium	Low	High	Low
3	Medium	Low	High	Low
4	Medium	Low	High	Low
5	Medium	Low	High	Low
6	Medium	Low	High	Low

Supplementary Table S11. The molecular docking results of compounds 1-6 against target proteins.

**Compound**	**Binding energy (kcal/mol)**	**Hydrogen bonds**	**Other bonds**
			**BCL-2**
**1**	-9.50	ASP B: 70	GLU B: 111, PHE B: 112, GLY B: 104, ALA B: 108, ARG B: 105, LEU B: 96, PHE B: 63, TYR B: 67, MET B: 74, PHR B: 71
**2**	-10.24	-	PHE B: 63, TYR B: 161, SER A: 76, GLY B: 162, LEU B: 160, ALA A: 72, PRO B: 163, GLU B: 58, ALA B: 59, ASP B: 62, VAL B: 107, GLY B: 104, TYR B: 67, ARG B: 66
**3**	-10.65	TYR B: 161	GLU B: 58, ALA B: 59, GLU B: 60, VAL B: 107, PHE B: 63, ARG B: 65, ASP B: 62, ASN A: 122, GLY B: 104, ARG B: 66, ARG A: 68, TRP B: 103, PRO B: 163, GLY B: 162, LEU B: 160, SER A: 76, GLU A: 73, ALA A: 72
**4**	-9.33	GLY B: 162, LEU B: 160	ALA A: 72, TYR B: 161, SER A: 76, PRO B: 163, THR B: 55, GLU B: 58, ALA B: 59, ASP B: 62, GLY B: 104, VAL B: 107, PHE B: 63
**5**	-10.90	PRO B: 163	GLU B: 58, TYR B: 161, ASP B: 62, PHE B: 63, GLY B: 104, ALA B: 59, VAL B: 107, ALA A:72, GLU A:73, LEU B: 160, GLY B: 162, SER A: 76

**6**	-10.55	-	TYR B: 67, VAL B: 107, PHE B: 63, ALA B: 59, ARG B: 66, ASP B: 62, GLU B: 58, GLU A: 119, ASN A: 122, SER A: 75, PRO B: 163, GLY B: 162, SER A: 76, LEU B: 160, ALA A: 72, ARG B: 105, GLY B: 104, ARG A: 68
			**BCL-XL**
**1**	-10.42	-	PHE A: 105, TYR A: 101, ARG A: 100, ASP B: 133, ARG A: 103, ARG B: 132, GLU B: 129, ALA A: 104, PHE B: 131, LEU B: 130, PHE B: 105, ALA B: 141, ASN B: 136, GLY B: 138, PHE B: 97
**2**	-11.39	-	TYR A: 195, TYR A: 101, GLU A: 96, ALA A: 93, VAL A: 141, PHE A: 97, PHE A: 105, ALA A: 142, ARG A: 139, ALA B: 104, LEU A: 108, ASN A: 136, GLY A: 138, TRP A: 137, LEU A: 130
**3**	-11.58	ARG B: 139	TYR A: 195, GLU A: 92, ASN A: 197, GGLU A: 96, ALA A: 93, VAL A: 141, GLY A: 94, GLY A: 138, PHE A: 97, TYR A: 101, ALA A: 142, ARG A: 139, ALA A: 104, PHE B: 105, ASM A: 136, PHE A: 105
**4**	-10.88	ARG A: 139	PHE A: 131, LEUA: 130, ALAB: 104, GLY1: 138, PHEA: 97, ALAA: 142, ASNA: 136, PHEA: 105, PHEB: 105, ARGB: 103, TYRB: 101, ARGA: 132, ARGA: 100, ASPA: 133, GLUA: 129
**5**	-11.61	-	SER B: 145, PHE B: 146, ARG B: 102, THR B: 109, LEU B: 108, SER B: 106, ALA A: 104, LEU B:130, GLU B: 129, TYR B: 101, ASP B: 133, ASN B: 136, PHE B: 97, GLY B: 138, ARG B: 139, PHE B: 105, ALA B: 142
**6**	-12.28	ARG B: 139	ARG A: 100, ARG A: 103, ASN B: 136, ALA A: 104, GLY B: 138, PHE B: 97, PHE B: 105, ASN A: 136, ARG B: 100, TYR B: 101, ALA A: 142, ALA B: 104, ARG A: 139, GLY A: 138, PHE A: 105, PHE A: 97, LEU A: 130, TYR A: 101, LEU B: 130, ARG A: 100
			**Caspase 3**
**1**	-9.09	TYRA: 197, PROB: 201	LYSA: 137, GLYB: 202, TYRB: 203, ALAB: 200, TYRA: 195, VALA: 266, META: 268, GLYA: 125, THRA: 140, ARGA: 164, LEUA: 136, GLUA: 124, ASPA: 135
**2**	-9.61	TYRB: 197	TYRA: 195, META: 268, PROB: 201, ARGA: 164, TYRA: 197, VALA: 266, VALB: 266, CYSA: 264, CYSB: 264, ARGB: 164, TYRB: 195, LEUB: 136, THRB: 140, GLYB: 125, GLYA: 202, LYSB: 137, GLUA: 124, GLUB: 124, PROA: 201
**3**	-10.28	TYRA: 197	TYRB: 197, VALA: 266, VALB: 266, META: 268, THRA: 140, ARGB: 164, TYRA: 195, LEUA: 136, GLYA: 125, ASPA: 135, LYSA: 137, TYRB: 203, GLUB: 167, GLYB: 202, GLUB: 124, GLUA: 124, PROB: 201, CYSA: 264, ARGA: 164, PROA: 201
**4**	-8.69	ASPB: 135, GLYB: 125, LYSB: 137, PROA: 201	GLUB: 124, ILEB: 126, GLYA: 202, GLUA: 124, ALAA: 200, TYRA: 203, METB: 268, TYRB: 195, THRB: 140, LEUB: 136, TYRB: 197
**5**	-9.22	PRO A: 201	GLU B: 124, GLY A: 202, GLU A: 124, MET B: 268, VAL B: 266, TYR B: 195, LEU B: 136, TYR B: 197, THR B: 140, LYS B: 137, ASP B: 135, GLY B: 125, ILE B: 126
**6**	9.03	-	LYS B: 137, LEU B; 136, ARG B: 164, PRO B: 201, VAL A: 266, ARG A: 164, TYR A: 197, GLU B: 124, GLU A: 124, ASP A: 135, GLY A: 125, LYS A: 137, VAL B; 266, PRO A: 201, GLY B: 125, TYR B: 197
			**Caspase 9**
**1**	-10.60	THRB: 181, PROB: 349	THRB: 179, ARGB: 178, HISB: 237, CYSB: 287, ARGB: 180, PHEB: 351, SERB: 361, PHEB: 348, GLYB: 350, GLYB: 360, GLYB: 182, ASPB: 186, SERB: 183, PROB: 357
**2**	-9.31	LYSB: 394, GLYB: 350	GLYB: 395, ILEB: 396, TYRB: 397, PHEB: 348, PROB: 349, LYSB: 398, PHEB: 351, CYSB: 287, GLYB: 288, TRPB: 354

**3**	-10.89	CYSB: 287	THRB: 179, THRB: 181, ARGB: 180, PHEB: 351, HISB: 237, GLYB: 288, TRPB: 354, ARGB: 355, GLYB: 350, PROB: 349, ARGB: 178, LYSB: 358
**4**	-9.63	ARGB: 180, THRB: 181, CYSB: 287	HISB: 237, PROB: 349, ARGB: 178, PHEB: 351, PROB: 357, THRB: 179, LYSB: 358, SERB: 183, ASPB: 186, SERB: 361, GLNB: 285, GLYB: 182, GLYB: 360,
**5**	-9.27	TRP B: 354	TYR B: 397, ILE B: 396, GLY B: 395, LYS B: 398, GLY B: 350, PHE B: 348, LYS B: 394, TRP B: 362, PRO B: 349, ARG B: 355, PHE B: 351, PRO B: 357
**6**	-11.31		ARG B; 355, TRP B: 354, GLY B: 350, PRO B: 349, PHE B: 351, GLY B: 288, HIS B: 237, CYS B: 287, ARG B: 180, GLY B: 182, GLY B: 360, ASP B: 186, SER B: 361, SER B: 183, THR B: 181, PRO B: 357, ARG A: 178, THR B: 179
			**CDK2**
**1**	-11.86	-	GLYA: 11, GLNA: 131, GLUA: 12, ASNA: 132, LYSA: 33, ASPA: 145, ALAA: 144, VALA: 18, PHEA: 80, ALAA: 31, VALA: 64, LEUA: 134, GLUA: 81, ILEA: 10, LEUA: 83, PHEA: 82, HISA: 84, GLNA: 85ASPA: 86
**2**	-12.50	LYSA: 33, ASPA: 145	LEUA: 83, PHEA: 82, GLUA: 81, ALAA: 31, VALA: 64, LEUA: 134, PHEA: 80, ALAA: 144, ASNA: 132, GLYA: 16, GLNA: 131, GLYA: 13, GLUA: 12, GLYA: 11, VALA: 18, ILEA: 10, ASPA: 86, GLNA: 85, LYSA: 89
**3**	-12.20	LYSA: 88	GLNA: 131, GLYA: 11, GLNA: 85, GLUA: 12, ALAA: 31, LEUA: 83, LEUA: 134, GLUA: 81, ALAA: 144, VALA.64, PHEA: 80, VALA: 18, LYSA: 9, PHEA: 82, HISA: 84, ASPA: 86, LYSA: 89, ASPA: 92
**4**	-12.53	LYSA: 33, ASPA: 145	LEUA: 83, GLUA: 81, ALAA: 31, LEUA: 134, VALA: 64, PHEA: 80, LEUA: 148, GLUA: 12, GLYA: 11, THRA: 14, GLYA: 13, VALA: 18, ASNA: 132, PHEA: 82, ALAA: 144, GLNA: 131, HISA: 84, ILEA: 10, ASPA: 86, GLNA: 85
**5**	-13.42	LYS A: 145, LYS A: 33	GLN A: 85, LEU A: 83, ILE A: 10, PHE A: 82, GLU A: 81, VAL A: 64, PHE A: 80, ALA A: 31, LEU A: 134, ALA A: 144, LEU A: 148, GLY A: 13, GLU A: 12, LYS A: 129, ASN A: 132, GLY A: 11, GLN A: 131, VAL A: 18, ASP A: 86
**6**	-10.90	LYS A: 89	GLY A: 11, LYS A: 88, ASP A: 92, ASP A: 86, GLN A: 131, LYS A: 9, HIS A: 84, LEU A: 298, PHE A: 82, LYS A: 20, LEU A: 83, GLN A: 85, LEU A: 134
			**CDK6**
**1**	-11.36	VAL A: 150	192LEU A: 32, PRO A: 35, LEU A: 34, ARG A: 30, ARG A: 82, ALA A: 149, ALA A: 152, LEU A: 151, SER A: 78, ILE A: 198, ALA A: 191, LEU A: 193, PRO A: 195, LEU A: 192, SER A: 194
**2**	-13.35	LEU A: 193	PRO A: 35, LYS A: 36, THR A: 38, CYS A: 85, LEU A: 34, SER A: 39, ALA A: 191, LEU A: 192, SER A: 194, GLU A: 31, PRO A: 195, LEU A: 151, VAL A: 150, ILE A: 198, LEU A: 185, VAL A;77, SER A: 78, ARG A: 52, LEU A: 40, ASP A: 81, TRP A: 41
**3**	-16.19	ARG A: 52	GLU A: 31, LEU A: 151, ILE A: 198, SER A: 194, ALA A: 152, PRO A: 195, LEU A: 193, PRO A: 74, LEU A: 185, VAL A: 77, ILE A: 190, ASP A: 81, ALA A: 191, LEU A: 192, THR A: 38, LEU A: 40, SER A: 39, TRP A: 41, ARG A: 82, SER A: 78, LYS A: 36, ALA A: 149, LEU A: 34VAL A: 150, LEU A: 32
**4**	-13.12	ALA A: 152, VAL A: 150	THR A: 38, LEU A: 192, LEU A: 193, LYS A: 36, SER A: 194, LEU A: 34, PRO A: 195, GLU A: 31, ALA A: 149, LEU A: 185, ILE A: 198, PRO A: 74, LEU A: 151, VAL A: 77, SER A: 78, LEU A: 40, ASP A: 81
**5**	-12.85	TRP A: 41, LEU A: 193	LEU A: 185, SER A: 194, ILE A: 198, ALA A: 152, PRO A: 195, VAL A: 77, SER A: 78, ARG A: 52, ALA A: 191, ASP A: 81, CYS A: 85, ARG A: 82, LEU A: 34, LEU A: 40, THR A: 38, LYS A: 36, LEU A: 192
**6**	-16.17	CYS A: 85	LEU A: 40, SER A: 39, THR A: 38, LYS A: 36, ARG A: 82, LEU A: 34, LEU A: 33, ARG A: 30, ALA A: 149, GLU A: 31, HIS B: 67, VAL A: 150, LEU A: 193, SER A: 194, LEU A: 151, PRO A: 195, ALA A: 152, ILE A: 198, LEU A: 185, VAL A: 77, SER A: 78, ILE A: 190, ARG A: 52, ASP A: 81, ALA A: 191, TRP A: 41, LEU A: 192

		**EGFR**	
**1**	-10.04	CYS A: 751	783ARG A: 817, CYS A: 773, GLY A: 772, LEU A: 694, MET A: 769, VAL A: 702, GLN A: 767, LEU A: 820, ALA A: 719, THR A: 830, MET A: 742, GLU A: 738, LYS A: 721, ASP A: 831
**2**	-10.27	ASP A: 831	LEU A: 694, GLY A: 772, MET A: 769, ALA A: 719, LEU A: 768, GLN A: 767, VAL A: 702, LEU A: 820, THR A: 830, THR A: 766, PHE A: 699, LYS A: 721, ARG A: 817, ASN A: 818, CYS A: 773, PHE A: 771
**3**	-10.59	-	VAL A: 702, ASP A: 831, ALA A: 698, GLU A: 734, ASP A: 813, ASN A: 818, ALA A: 731, LEU A: 834, GLY A: 833, ARG A: 817, LYS A: 851, LEU A: 723, ILE A: 735, GLU A: 738, PHE A: 699, LYS A: 721
**4**	-10.62	THR A: 766, ARG A: 817	VAL A: 702, GLN A: 767, ALA A: 719, LEU A: 768, MET A: 769, LEU A: 694, LEU A: 820, GLY A: 772, CYS A: 773, ASN A: 818, ASP A: 831, PHE A: 699, LYS A: 721, THR A: 830, GLU A: 738, MET A: 742, CYS A: 751
**5**	-10.73	ASP A: 831	LYS A: 721, ASN A: 818, PHE A: 699, ARG A: 817, LEU A: 820, CYS A: 773, PHE A: 771, VAL A: 702, LEU A: 694, MET A: 769, GLY A: 772, GLN A: 767, LEU A: 768, ALA A: 719, THR A: 766, THR A: 830
**6**	-10.02	-	ALA A: 719, GLY A: 772, VAL A: 702, THR A: 766, LYS A: 721, ASP A: 831, PHE A: 699, GLY A: 695, ASN A: 818, ARG A: 817, CYS A: 773, LEU A: 820, LEU A: 768, MET A: 769, LEU A: 694
		**VEGFR**	
**1**	-10.25	ARG A: 1049, ASN A: 921, LEU A: 838, CYS A: 917	GLY A: 839, VAL A: 846, ALA A: 864, GLU A: 915, PHE A: 916, LYS A: 918, PHE A: 919, GLY A: 920, LEU A: 1033, PHE A: 1045
**2**	-11.56		PHE A: 843, LYS A: 866, ALA A: 879, LEU A: 880, GLU A: 883, ILE A: 886, LEU A: 887, ILE A: 890, VAL A: 896, VAL A: 897, LEU A: 1017, CYS A: 1022, ILE A: 1023, HIS A: 1024, ARG A: 1025, ILE A: 1042, CYS A: 1043, ASP A: 1044, GLY A: 1046
**3**	-11.30	-	HIS A: 814 ,CYS A: 815 ,LYS A: 866 ,SER A: 882 ,GLU A: 883 ,ILE A: 886 ,LEU A: 887 ,ILE A: 890 ,VAL A: 896 ,VAL A: 897 ,LEU A: 1017 ,CYS A: 1022 ,ILE A: 1023 ,HIS A: 1024 ,ARG A: 1025 ,ASP A: 1026 ,ILE A: 1042 ,CYS A: 1043 ,GLY A: 1046
**4**	-10.78	ILE A: 1023	PHE A: 843, LYS A: 866, ALA A: 879, LEU A: 880, ILE A: 886, LEU A: 887, ILE A: 890, VAL A: 896, VAL A: 897, LEU A: 1017, CYS A: 1022, HIS A: 1024, ARG A: 1025, ILE A: 1042, CYS A: 1043, ASP A: 1044, GLY A: 1046
**5**	-12.29	-	ILE A: 886, GLU A: 883, ASP A: 1044, GLY A: 1046, VAL A: 914, PHE A: 1045, CYS A: 1043, LEU A: 887, VAL A: 897, ILE A: 1042, VAL A: 896, ILE A: 890, LEU A: 1017, HIS A: 1024, ILE A: 1023, CYS A: 1022, ARG A: 1025
**6**	-10.86	-	SER A: 882, ILE A: 886, HIS A: 1024, ARG A: 1025, HIS A: 889, HIS A: 814, VAL A: 897, LEU A: 1017, CYS A: 1022, ILE A: 890, ASP A: 1044, VAL A: 896, CYS A: 1043, LEU A: 887, LEU A: 880, GLU A: 883, ALA A: 879, PHE A: 843, GLY A: 1046
		**P53**	
**1**	-9.86	GLN A: 23	ARG A: 10, PHE A: 16, ASN A: 17, LYS A: 20, ILE A: 21, ILE A: 22, GLY A: 24, GLU A: 89, TYR A: 92, LEU A: 100, LEU A: 103, ARG A: 104, CYS A: 114, PRO A: 115
**2**	-10.39	GLNA: 23, ILEA: 21	ARG A: 10, PHE A: 16, ASN A: 17, LYS A: 20, ILE A: 22, GLU A: 89, ARG A: 90, TYR A: 92, LEU A: 100, LEU A: 103, ARG A: 104, CYS A: 114, PRO A: 115, ARG A: 203

**3**	-10.20	ARG A:203, SER A: 204, TYR A: 92	ARG A: 10, ASN A: 17, LYS A: 20, ILE A: 21, ILE A: 22, GLN A: 23, ARG A: 90, LEU A: 100, LEU A: 103, ARG A: 104, CYS A: 114, PRO A: 115, LEU A: 125, ALA A: 200, SER A: 228, VAL A: 229, THR A: 230
**4**	-10.83	ILE A: 21, PHE A:16, THR A: 230, ARG A: 203	ARG A: 10, ASN A: 17, LYS A: 20, ILE A: 22, GLN A: 23, GLU A: 89, TYR A: 92, LEU A: 100, GLY A: 199, ALA A: 200, VAL A: 229, PRO A: 231, ASN A: 232
**5**	-11.44	ARG A: 104, GLN A: 23	ALA A: 200, PHE A: 16, ILE A: 22, ILE A: 21, ASN A: 17, LEU A: 100, LYS A: 20, CYS A: 114, PRO A: 115, LEU A: 103, GLY A: 24, ARG A: 203, ARG A: 10, GLY A: 199, GLU A: 89
**6**	-11.58	ARG A: 10, ARG A: 203	ASN A: 232, GLU A: 13, LYS A: 20, PHE A: 16, GLN A: 23, ILE A: 21 ILE A: 22, LEU A: 100, PRO A: 115, CYS A: 114, ARG A: 90, LEU A: 103, PHE A: 117, TYR A: 92, ALA A: 200, SER A: 228, GLU A: 89, VAL A: 229, THR A: 230, GLY A: 199, ASN A: 17, LYS A: 18
			**PARP-1**
**1**	-11.15	GLY A: 863	GLY A: 888, TYR A: 889, HIS A: 862, SER A: 904, ALA A: 898, LYS A: 903, PHE A: 897, TYR A: 896, GLU A: 988, MET A: 980
**2**	-11.32	ALA A: 880	ASN A: 868, PRO A: 881, ARG A: 878, ILE A: 879, ILE A: 895, GLY A: 894, TYR A: 896, TYR A: 889, HIS A: 862, GLN A: 875, LUE A: 877, GLY A: 876, GLY A: 871, ILE A: 872
**3**	-13.59	ARG A: 878	GLN A: 875, GLY A: 876, GLY A: 871, LEU A: 877, ILE A: 872, SER A: 864, HIS A: 862, ASN A: 868, HIS A: 909, ARG A: 865, GLY A: 863, PHE A: 897, GLU A: 988, LYS A: 903, ASN A: 897, ALA A: 898, SER A: 904, TYR A: 896, TYR A: 907, GLY A: 888, TYR A: 889, GLY A: 894, ALA A: 880
**4**	-11.42	GLY A: 876, ARG A: 878, ALA A: 880	PRO A; 881, ILE A: 895, ILE A: 879, GLY A: 894, TYR A: 896, TYR A: 889, HIS A: 862, ILE A: 872, LEU A: 877, GLY A: 871, GLN A: 875, ASN A: 868
**5**	-12.97	GLY A: 894	SER A: 904, ALA A: 898, GLU A: 988, LYS A: 903, MET A: 890, TYR A: 896, GLY A: 888, TYR A: 889, LYS A: 893, ALA A: 880, ILE A: 879, ARG A: 878, ILE A: 895, LEU A: 877, HIS A: 862, PHE A: 897
**6**	-13.39	TYR A: 889, ALA A: 880	ARG A: 878, LEU A: 877, ILE A: 872, ILE A: 895, GLY A: 894, HIS A: 862, TYR A: 896, SER A: 864, GLY A: 863, LYS A: 903, ALA A: 898, GLU A: 988, PHE A: 897, TYR A: 907, SER A: 904, GLY A: 888, ILE A: 879, PRO A: 881
